# Subwavelength control of light transport at the exceptional point by non-Hermitian metagratings

**DOI:** 10.1126/sciadv.adf3510

**Published:** 2023-05-12

**Authors:** Yihao Xu, Lin Li, Heonyeong Jeong, Seokwoo Kim, Inki Kim, Junsuk Rho, Yongmin Liu

**Affiliations:** ^1^ Department of Mechanical and Industrial Engineering, Northeastern University, Boston, MA 02115, USA.; ^2^ Department of Mechanical Engineering, Pohang University of Science and Technology (POSTECH), Pohang 37673, Republic of Korea.; ^3^ Department of Biophysics, Institute of Quantum Biophysics, Sungkyunkwan University, Suwon 16419, Republic of Korea.; ^4^ Department of Intelligent Precision Healthcare Convergence, Sungkyunkwan University, Suwon 16419, Republic of Korea.; ^5^ Department of Chemical Engineering, Pohang University of Science and Technology (POSTECH), Pohang 37673, Republic of Korea.; ^6^ POSCO-POSTECH-RIST Convergence Research Center for Flat Optics and Metaphotonics, Pohang 37673, Republic of Korea.; ^7^ National Institute of Nanomaterials Technology (NINT), Pohang 37673, Republic of Korea.; ^8^ Department of Electrical and Computer Engineering, Northeastern University, Boston, MA 02115, USA.

## Abstract

The concept of non-Hermitian physics, originally developed in the context of quantum field theory, has been investigated on distinct photonic platforms and created a plethora of counterintuitive phenomena. Interfacing non-Hermitian photonics and nanoplasmonics, here, we demonstrate unidirectional excitation and reflection of surface plasmon polaritons by elaborately designing the permittivity profile of non-Hermitian metagratings, in which the eigenstates of the system can coalesce at an exceptional point. Continuous tuning of the excitation or reflection ratios is also possible through altering the geometry of the metagrating. The controllable directionality and robust performance are attributed to the phase transition near the exceptional point, which is fully confirmed by the theoretic calculation, numerical simulation, and experimental characterization. Our work pushes non-Hermitian photonics to the nanoscale regime and paves the way toward high-performance plasmonic devices with superior controllability, performance, and robustness by using the topological effect associated with non-Hermitian systems.

## Teaser

Non-Hermitian meta-gratings enable unidirectional excitation and reflection of optical surface waves at the nanoscale.

## INTRODUCTION

Non-Hermitian systems, which are open systems that can exchange energy, matter, or information with their surroundings by engineering the distribution of gain or loss materials, have shown notable phenomena since the seminal work by Bender *et al.* (*1*, *2*). It is commonly accepted in quantum mechanics that Hamiltonians need to be Hermitian to produce real eigenvalues. However, if the spatially varying complex penitential energy $V(\vec{r})$ in a system satisfies $V(\overset{⇀}{r})=V^{*}(-\overset{⇀}{r})$, implying that the non-Hermitian Hamiltonian obeys parity-time (*PT*) symmetry, then entirely real eigenvalue spectra can exist under certain thresholds, known as exceptional points (EPs). Because of the equivalence of the mathematic form between the Schrödinger equation and the paraxial equation of diffraction in optics, people have demonstrated that photonic systems can serve as an alternative platform to study non-Hermitian physics. Since the refractive index acts as an equivalent “optical potential” in the paraxial equation, the requirement for a *PT-*symmetric photonic system then becomes $n_{r}(\overset{⇀}{r})=n_{r}(-\overset{⇀}{r})$ and $n_{i}(\overset{⇀}{r})=-n_{i}(-\overset{⇀}{r})$, where *n_r_* and *n_i_* are the real and imaginary parts of the refractive index, respectively. Many intriguing phenomena and applications in non-Hermitian photonics have emerged, including controllable mode selection (*3*–*5*), singularity-enhanced sensing (*6*–*10*), robust mode propagation (*11*–*13*), waveguide mode control (*14*–*17*), non-Hermitian induced topology (*18*–*24*), temporal photonic lattices (*25*), transient growth and dissipation of waves (*26*, *27*), and stable single-mode lasers as well as perfect absorbers (*28*–*34*). However, most of the reported works on non-Hermitian photonics have relied on elements with dimensions comparable to or larger than the wavelength, including microring resonators and coupled waveguides (*4*, *8*, *28*, *30*, *35*), which are not desirable for device miniaturization and integration purpose. On the other hand, it remains challenging to implement subwavelength building blocks for non-Hermitian systems, and there are only a handful reported works along this direction (*36*–*38*). The major hurdle lies in the increased sensitivity near the EPs in non-Hermitian systems (*7*, *8*, *10*). It means that the EPs are difficult to reach and maintain unless we can have precise control of both the design parameters and the ambient conditions.

In parallel to non-Hermitian photonics, plasmonics has drawn considerable attention in the optics community because of the competence of surface plasmon polaritons (SPPs) to surpass the diffraction limit and enhance light-matter interactions (*39*–*43*). Owing to the two advantages, plasmonics has been applied in many areas, including spectroscopy (*44*), sensing (*45*), amplifiers and lasers (*46*), and nanolithography (*47*). In particular, it is envisioned that plasmonics is a promising candidate for the next-generation optical circuit (*48*, *49*). In this regard, it is of great importance to achieve controllable manipulation of the SPPs with superior directionality and robustness. At the early stage, nanoslits (*50*–*53*), apertures (*54*), and nanoantennas (*55*, *56*) were used to compensate for the momentum mismatch, and at the same time, the interference of plasmon waves from these nanostructures can generate SPPs propagating along the selected direction. However, without periodic patterning, the scattering loss of the isolated nanostructures could be severe. Recently, the emergence of metasurfaces enlightens new possibilities in the manipulation of SPPs (*57*, *58*). Metasurfaces can achieve high performance in terms of controllability, while the directional excitation of SPPs is usually manipulated by the polarization of the incident light. However, the meta-atoms, the building blocks of metasurfaces, need to be patterned as a two-dimensional (2D) array, which unavoidably suffers from a high loss because of the increasing number of meta-atoms and, in the meantime, raises the fabrication difficulties.

Here, we propose and demonstrate subwavelength non-Hermitian metagratings that enable unidirectional control of SPPs at the EPs, by synergistically interfacing non-Hermitian physics and nanoplasmonics. Our approach, in contrast to most of the published works, relies on the *PT* symmetry criterion to guide the subwavelength design and control of multiple elements inside each unit cell. Our design not only provides large degrees of freedom for manipulating SPP excitation but also guarantees the robustness of the unidirectionality due to the characteristics of EPs. The merging of non-Hermitian physics and nanoplasmonics has profound impacts. First of all, the large flexibilities of structural design enabled by plasmonics can provide exciting opportunities to realize subwavelength synthetic test beds to study non-Hermitian physics in the nanoscale regime. For instance, we can readily design plasmonic nanostructures with complex permittivity modulation and desired coupling to construct a non-Hermitian system, so that SPPs can be modulated in unusual ways as we will discuss in this paper. Second, the strategy of gain and loss manipulation based on *PT* symmetry in non-Hermitian physics could help to address the long-standing issue of energy dissipation in plasmonics, using the lossy components for mode selection (*3*, *28*, *35*). The orthogonality of the eigenmodes gradually breaks during the continuous transition from the *PT*-symmetric phase to the *PT*-broken phase, along with a complex pair of eigenenergies, one of which corresponds to a high-loss state while the other corresponds to a low-loss or even amplified state. Therefore, we can potentially transform the undesired lossy plasmonic structures into superior optical elements with substantially improved efficiency. Last but not the least, disorder, defects, and environmental variations, which often exist in any practical systems, strongly influence the response and performance of plasmonic nanostructures. The topological states in non-Hermitian systems (*20*, *59*) manifest the potential to address this issue. Ultimately, we may develop integrated plasmonic devices with low power consumption, small footprint, and robust performance for optical information processing, secure communications, enhanced sensing, and other applications.

## RESULTS

**Fig. 1.**
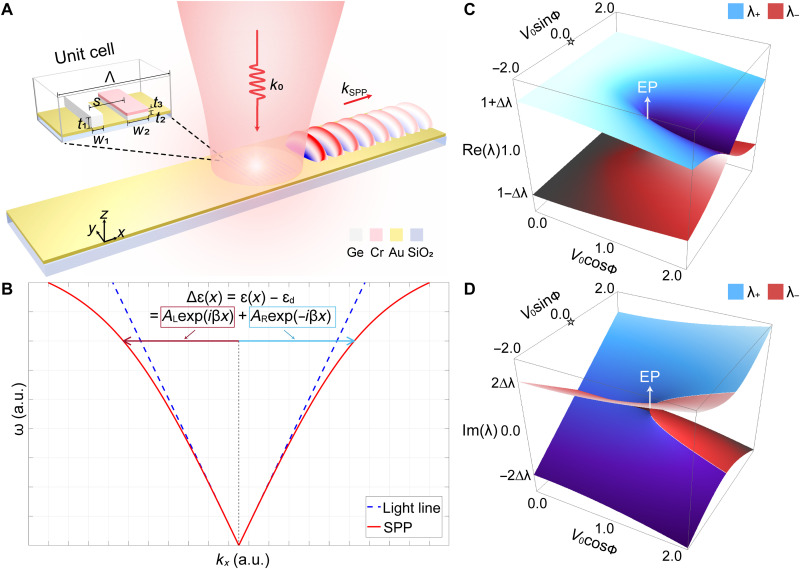
Unidirectional excitation of SPPs at the EP. (**A**) Schematic of the metagrating for unidirectional excitation of SPPs. (**B**) Mechanism of controllable excitation of SPPs. The two first-order Fourier components of the permittivity profile contribute to the excitation of SPPs along the ±*x* direction. Real (**C**) and imaginary (**D**) parts of the eigenvalues of the two eigenmodes of SPPs in a 2D polar parameter space (*X* = *V*_0_ cos ϕ, *Y* = *V*_0_ sin ϕ), in which the two eigenvalues are represented by the blue and red sheets, respectively. One EP occurring at (*V*_0_, ϕ) = (1,0) is marked in the figure. The origin of the polar plot (at *V*_0_ = 0) is indicated by the star symbol.

The first example of non-Hermitian plasmonics that we want to show is unidirectional excitation of SPPs when linearly polarized light is normally incident on a non-Hermitian metagrating, as illustrated in Fig. 1A. By designing the geometric parameters and the spatial distribution of permittivity (ε = ε*_r_* − *i*ε*_i_*) within one unit cell of the grating, a passive *PT* symmetry can be approximated. The underlying mechanism is attributed to the EP behavior in the non-Hermitian system. To start with, let us consider an ideal metal-dielectric interface where the dielectric layer has the following permittivity profile

$$\varepsilon (x)=\varepsilon_{d}+A[\cos\beta x-iV_{0}\sin(\beta x-\phi )]=\varepsilon_{d}+A_{L}\exp(i\beta x)+A_{R}\exp(-i\beta x)$$ (1)

Here, ε_d_ is the background permittivity, *V*_0_ is the relative modulation amplitude of the imaginary part of the permittivity, β is the wave number of the SPPs, ϕ describes an additional phase shift of the imaginary modulation, and *A* represents the perturbation strength that is much smaller than ε_d_ (i.e., *A* ≪ ε_d_). We start with this ideal system for the following reasons: First, such an ideal setting for permittivity distribution has an analytical and simple mathematical form while keeping the periodic and weakly perturbed properties of a practical grating system. We therefore can theoretically examine how this non-Hermitian system behaves in the parameter space because the ideal platform makes it simple to change the parameters (*V*_0_ and ϕ). The modulation of the permittivity, which is treated as a perturbation to the background permittivity ε_d_, can be decomposed to the two Fourier components at the first order with coefficient given by *A*_R/L_. Apparently, when ϕ = 0 or π, ε(*x*) = ε^*^(−*x*) is satisfied, ensuring the *PT* symmetry in this system. The dispersion diagram of the propagating SPPs is depicted in Fig. 1B. By solving Maxwell’s equations, we can find that the magnetic field amplitude of the excited SPPs along the ±*x* direction (*H*_R/L_) is linearly proportional to *A*_R/L_ (see note S1), that is

$$\left|\frac{H_{R}}{H_{L}}\right|=\left|\frac{A_{R}}{A_{L}}\right|$$ (2)

Therefore, we conclude that *A*_R/L_ contributes to the momentum compensation for the SPPs propagating to the right/left side (+*x*/−*x* direction), as indicated by the blue and red arrows in Fig. 1B, respectively. When *A*_R_ or *A*_L_ equals zero, which corresponds to the EPs of the system (see note S2), an ideal unidirectional excitation of SPPs can be achieved.

By varying the perturbation strength of the imaginary modulation (*V*_0_) and the additional phase shift of the imaginary modulation (ϕ), we can theoretically predict the complex eigenvalues (i.e., $\lambda \equiv \omega /{\tilde{\omega }}_{0}$ as defined in eq. S20) of the two SPPs eigenmodes. The real and imaginary parts of eigenvalues, as a function of *V*_0_ and ϕ, are presented in a 2D polar parameter space as shown in Fig. 1 (C and D). In both plots, the value of *V*_0_ (varying from 0 to 2) is denoted by the distance from the origin (indicated by the star symbol), and the value of ϕ is denoted by the azimuthal angle. The two eigenvalues $\lambda_{+}=\omega_{+}/{\tilde{\omega }}_{0}$ and $\lambda_{-}=\omega_{-}/{\tilde{\omega }}_{0}$ are represented by the blue and red sheets in the plot. The value of Δλ in the plot, as defined in eq. S20, is linearly related to the perturbation strength *A*. Only a half-space [ϕ ∈ (−π/2, π/2)] is plotted because of the mirror symmetry along the *V*_0_ cos ϕ axis. The simultaneous degeneration of the eigenvalues in both plots indicates the existence of an EP at (*V*_0_, ϕ) = (1, 0) (i.e., *V*_0_ cos ϕ = 1 and *V*_0_ sin ϕ = 0). Note that the two eigenmodes of the SPPs coalesce at the EP, corresponding to unidirectional SPPs mode toward the +*x* direction, while the other EP occurs at (*V*_0_, ϕ) = (1, π) (in the other half-space), corresponding to unidirectional SPPs toward the −*x* direction. We use the polar space considering the periodic nature of the parameter ϕ, so that the feature in the vicinity of the EPs can be better visualized. The EP behavior in 1D parameter space (by sweeping *V*_0_ or ϕ) is also calculated, and the results are presented in note S2.

Despite being mathematically simple, the ideal system is not practical for fabrication. It requires a continuous *PT*-symmetric permittivity profile with balanced gain and loss, which is extremely challenging if not impossible in practice. Therefore, we choose a design of metagratings consisting of discrete nanostrips, which can approximate the passive version of the *PT*-symmetric distribution. As a result, unidirectional excitation of SPPs also occurs in such a non-Hermitian system although a strict *PT* symmetry is not preserved. We generate the design candidates with a high *A*_R_/*A*_L_ ratio based on the methodology described in note S3. Then, after considering all practical factors, we choose the design with the highest field contrast using full-wave modeling, in which the scattering and back-reflection of each grating are taken into account. The designed and optimized geometric parameters of the unit cell are shown in the inset of Fig. 1A. The widths of two nanostrips are *w*_1_ = 83 nm and *w*_2_ = 162 nm, and the heights of each layer are *t*_1_ = 80 nm, *t*_2_ = 30 nm, and *t*_3_ = 22 nm. The center-to-center separation distance between the two nanostrips is *s* = 280 nm. This quarter-period shift corresponds to the required phase shift between the peak of real [i.e., cos(·)] and imaginary [i.e., sin(·)] modulations at the EP when ϕ = 0°. The entire metagrating array consists of nine unit cells in total. The period is set as Λ = 1140 nm, which is designed to compensate the momentum mismatch between free-space wave and SPPs according to the following formula

β=Re(k0εairεgoldεair+εgold)=2πΛ (3)

Here, $k_{0}=\frac{2\pi }{\lambda_{0}}$ is the wave number of the light in free space, and the wavelength is set at λ_0_ = 1150 nm. Note that the excitation of the SPPs can be modified as the separation distance *s* changes, because it essentially provides an additional phase term in the imaginary modulation, given by $\phi =\frac{2\pi s}{\text{Λ}}-\frac{\pi }{2}$. Consequently, we can continuously change the ratio between ∣*A*_R_∣ and ∣*A*_L_∣, as discussed in detail in the following.

**Fig. 2.**
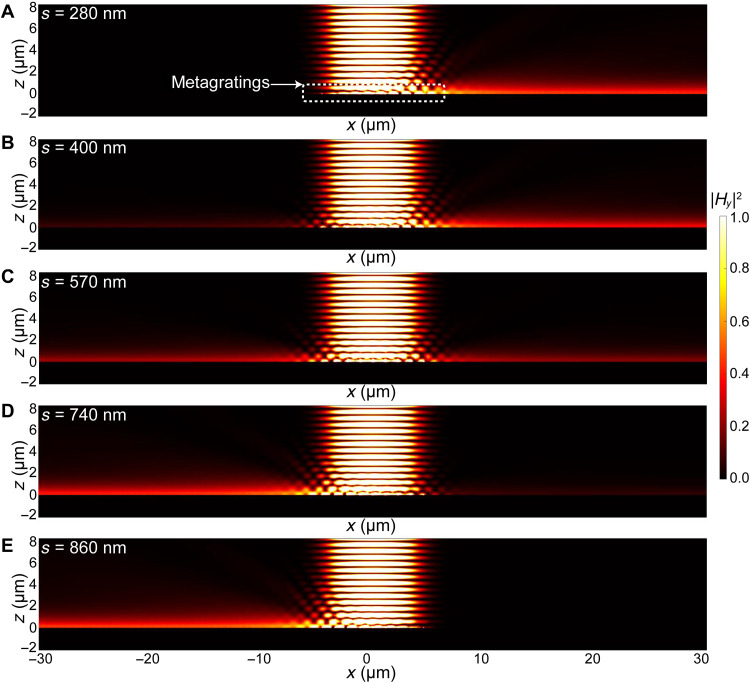
Numerical simulation of SPP excitation in metagratings. Simulated magnetic field intensity |*H_y_*|^2^ as the separation distance between the two nanostrips in the unit cell of metagratings is varied: (**A**) *s* = 280 nm, (**B**) *s* = 400 nm, (**C**) *s* = 570 nm, (**D**) *s* = 740 nm, and (**E**) *s* = 860 nm.

We have performed full-wave simulations using the commercial software COMSOL Multiphysics to validate the expected performance of the device. In the 2D simulation, the incident light is set as a Gaussian beam with *x*-polarization at λ_0_ = 1150 nm, which covers the entire metagrating array. In Fig. 2A, we plot the intensity of the transverse magnetic field component (∣*H_y_*∣^2^). We can observe unidirectional excitation of SPPs along the +*x* direction for the designed geometry *s*_R_ = 280 nm. The conversion efficiency from the incident light to SPPs is about 6.8% based on our numerical simulations. The extinction ratio (defined as ∣*H*_R_/*H*_L_∣) observed in the simulation is ≫10. When the separation distance between two nanostrips in one unit cell is increased from 280 to 860 nm, ϕ changes from 0 to π. We actually go along a circular trajectory, with the star symbol as the center (*V*_0_ = 1, ϕ = 0 → π), from one EP to the other EP in the 2D parameter space. The excitation of SPPs gradually becomes symmetric at *s* = 570 nm (corresponding to ϕ = π/2), and finally, SPPs are steered to the −*x* direction at *s* = 860 nm (corresponding to ϕ = π), as shown in Fig. 2 (B to E). Note that at *s*_L_ = Λ − *s*_R_ = 860 nm, the unit cell just becomes the mirror image of the originally designed one, which also explains the mirror symmetry of the 2D parameter space. We conduct another set of simulations on a Hermitian grating system (see note S4). In this case, we cannot observe the high-contrast unidirectional excitation of SPPs at any separation distance *s*, which verifies the necessity and significance of the imaginary modulation to achieve the EPs in a non-Hermitian system. Moreover, the Hermitian system may produce high diffraction to the free space and exhibit unstable excitation efficiency when the shift is varied. Further investigation has confirmed that a transition from a trivial (zero winding number) to a nontrivial (nonzero winding number) topological phase takes place as the system evolves along the trajectory from the Hermitian system (*V*_0_ = 0) to the EPs of the non-Hermitian system. The unidirectionality of SPPs in the non-Hermitian metagrating, which emerges at the topological transition points, is robust to the permittivity perturbation, which often occurs because of fabrication imperfection. In particular, the excitation contrast of SPPs remains almost unchanged when a single unit cell is perturbed and is three times more robust than the Hermitian counterpart when multiple unit cells are perturbed (see note S5). Therefore, the designed non-Hermitian metagratings can serve as a device to selectively control SPP propagation with user-defined field contrast and pronounced robustness. We summarize the values of ∣*A*_R_/*A*_L_∣ and ∣*H*_R_/*H*_L_∣ in Table 1, when *s* varies from 200 to 900 nm. These two ratios are always close to each other, and agree well with our theoretical expectation. It is worth pointing out that our approach to achieving unidirectional SPPs is fundamentally different from the previous works. Our design approach relies on the local control of multiple elements within a subwavelength unit cell, under the guidance of the global *PT* symmetry requirement of the complex dielectric constant, rather than the optimization of scattering units and thus the relative phases between them (*54*, *55*, *58*), or the near-field interference of a single radiative source (*53*). Because of the sufficient degrees of freedom in the design, we can obtain the on-demand manipulation of SPPs while maintaining the device performance in the presence of perturbation.

**Table 1.** Ratios of Fourier components and magnetic fields of SPPs in metagratings. The ratios of the first-order Fourier components (calculation) and ratios of magnetic field amplitude of SPPs in two directions (simulation) when the separation distance *s* is varied.

*s* (nm)	200	280	300	400	500	600	700	800	860	900
∣*A*_R_/*A*_L_∣	3.81	≫10	10.71	3.02	1.49	0.85	0.46	0.17	≪0.1	0.16
∣*H*_R_/*H*_L_∣	3.29	≫10	11.08	2.09	1.20	0.81	0.51	0.20	≪0.1	0.16

**Fig. 3.**
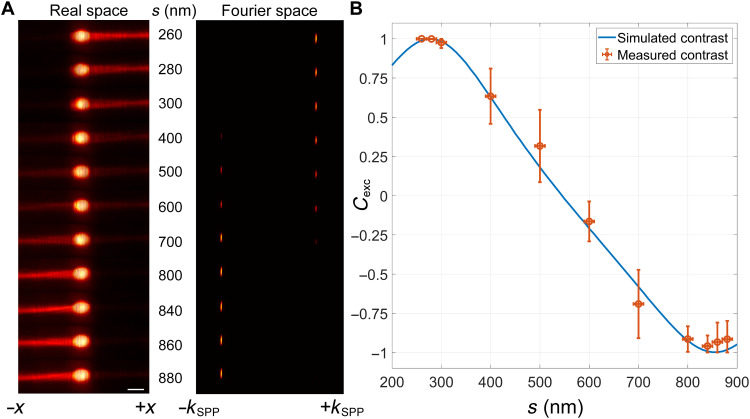
Characteristics of the excited SPPs in metagratings. (**A**) Real space and Fourier space images of SPPs characterized by leakage radiation microscopy. Scale bar, 10 μm. (**B**) Simulated and measured contrast of the excited SPPs.

We have performed experiments to validate our design. After fabricating the non-Hermitian metagratings by two-step electron beam lithography alignment, we characterize their performance using leakage radiation microscopy. The details of the fabrication and optical setup for the measurement are discussed in notes S6 and S7. The characteristic of excited SPPs can be observed in either real space (Fig. 3A, left) or the Fourier space where SPPs propagating to each direction are projected to a tiny arc in the momentum space (Fig. 3A, right). From both the real space and Fourier space images, we can see SPPs propagating only along the +*x* direction when distance *s* is around 280 nm. When *s* gradually increases, the relative magnitude of the excited SPPs propagating along the −*x* direction also increases. In addition, at *s* = 860 nm, the excited SPPs reverse the propagation direction to −*x*. All the results are in excellent agreement with our design and numerical simulations. To better characterizing the directional control performance of SPP excitations, we can define the contrast of the excited SPPs as

$$C_{\mathrm{exc}}=\frac{I_{r}-I_{l}}{I_{r}+I_{l}}$$ (4)

where *I*_r_ and *I*_l_ are the intensity of the excited SPPs propagating along the +*x* and −*x* directions, respectively. The value of the contrast will be +1 (−1) when an ideal propagating SPPs are excited to the +*x* (−*x*) direction. The detailed method to extract the intensity from the measurement is discussed in note S8. The measured and simulated contrasts with respect to *s* are plotted in Fig. 3B, which agree with each other very well. It is apparent that the non-Hermitian metagrating can achieve tunable selective excitation of SPPs.

**Fig. 4.**
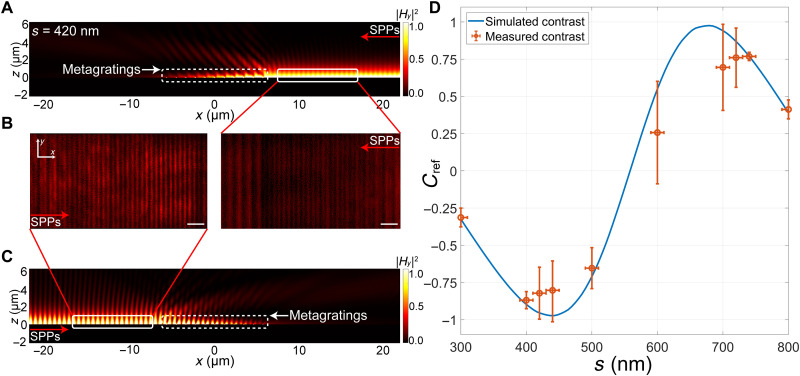
Characteristics of the reflected SPPs in metagratings. Simulated |*H_y_*|^2^ fields of the interference patterns when SPPs are incident from (**A**) the right and (**C**) the left side of the metagratings. (**B**) Measured interference patterns of SPPs captured by a CCD camera for *s*_R_ = 420 nm, which corresponds to the interference patterns within the area indicated by white solid lines in (A) and (C). Scale bars, 2 μm. (**D**) Simulated and measured contrast of the reflected SPPs.

We further explore how to manipulate the reflection of SPPs based on non-Hermitian metagratings, which is also important for integrated plasmon devices. Specifically, we demonstrate a metagrating to strongly reflect SPPs when SPPs are incident on the grating from one side (e.g., left side), while a much weaker reflection occurs when the SPPs illuminate from the other side (e.g., right side). In other words, the non-Hermitian metagrating behaves as a one-way invisible reflector. To realize the reflection control of the SPPs, we need to consider the second-order Fourier component (i.e., *A*_2L_ and *A*_2R_) of the permittivity profile. The magnitude of the additional momentum that requires to reflect SPPs is 2β (from ±β to ∓β), and a straightforward way to achieve this is to halve the periodicity of the grating array to $\text{Λ}=\frac{2\pi }{2\beta }=570\;\mathrm{nm}$ and then adjust the geometric parameter of the nanostrips. However the periodicity will be too small to contain two nanostrips with an optimized separation distance in this case. Therefore, we keep the periodicity to be Λ = 1140 nm because it can also provide an additional momentum of 2β and increase the separation distance between nanostrips to 3Λ/8 (see note S9). This design not only improves the fabrication tolerance but also provides more degrees of freedom in the design process. The other geometric parameters optimized in the calculation and simulation are the following: *w*_1_ = 130 nm, *w*_2_ = 130 nm, *t*_1_ = 48 nm, *t*_2_ = 26 nm, and *t*_3_ = 16 nm. The separation distance between the two nanostrips in one unit cell is set as *s*_R_ = 420 nm, which is close to 3Λ/8 and further optimized in simulation, to ensure *A*_2L_ ≫ *A*_2R_. The simulation results of the unidirectional reflection are shown in Fig. 4 (A and C). When the SPPs are reflected by the designed non-Hermitian metagrating, the incident and reflected surface waves will interfere in the overlap region, and a variation of the intensity caused by the standing wave pattern can be observed. In Fig. 4A, the SPPs are incident from the right, and we can only see a weak standing wave pattern, indicating low reflectance. The situation is different in Fig. 4C, when the SPPs are incident from the left. We can observe a strong standing wave pattern representing a high reflectance of SPPs. Similar to the previous device, we can vary *s* to continuously tune the reflection intensity of SPPs.

To verify the unidirectionality of this design, we carry out the experimental measurement. We use a grating coupler made of only Ge to evenly excite SPPs on both sides. Two identical metagratings are placed on the left and right sides of the grating coupler, respectively, with a 45-μm separation distance to the coupling grating (see note S10). The interference patterns of the incident and reflected SPPs are captured using a charge-coupled device (CCD) camera for different samples with varying separation distances *s*. Figure 4B shows the measured field intensity at *s*_R_ = 420 nm. We can observe a pronounced interference pattern when the SPP illuminates from the left, which indicates a strong reflection. The interference is much weaker when illuminating the SPPs from the right, indicating a low reflection. The situation is reversed at *s*_L_ = 720 nm, where the unit cell is the mirror image as *s*_R_ = 420 nm. All the observations are consistent with the simulation results in Fig. 4 (A and C). We then analyze the image in a more quantitative manner. From the real space image taken by the CCD camera, we can extract the exact reflectance of SPPs. The contrast of the reflectance is defined as

$$C_{\mathrm{ref}}=\frac{R_{r}-R_{l}}{R_{r}+R_{l}}$$ (5)

where *R*_r_, *R*_l_ are the reflectance of the SPPs when SPPs illuminate from the +*x* and −*x* direction, respectively. In Fig. 4D, we plot the measured contrast and the simulated contrast of reflectance, showing good agreement with each other. Moreover, the slight deviation of the measured contrast from the simulation results is attributed to both the fabrication imperfection of the metagratings and the coherent background intensity induced by the laser excitation (*60*).

## DISCUSSION

On the basis of the experimental results measured in two different metagrating systems, we demonstrate on-demand control of SPPs by elaborately designed non-Hermitian metagratings. When the permittivity profile of the dielectric medium approximates the *PT* symmetry, robust unidirectional excitation of SPPs with high contrast can be observed at EPs where the nontrivial topology occurs. The excitation ratio of SPPs on both sides can be continuously tuned by the separation distance between nanostrips in the unit cell. Furthermore, one-way reflection of SPPs is experimentally demonstrated. Our results show that the rich non-Hermitian physics can be adopted to generate, harness, and transport light and energy at the nanoscale in an unprecedented manner. By further investigating and taking advantage of the topological features of non-Hermitian systems, we could potentially develop defect-immune and high-performance plasmonic devices for a wide range of applications.

## MATERIALS AND METHODS

### Numerical simulations

The full-wave simulations for the excitation and reflection of SPPs were performed using COMSOL Multiphysics, a commercial software that uses the finite element method. At the top boundary, a scattering boundary condition with incident light in a Gaussian profile was defined. Perfectly matched layers were applied to the left, right, and bottom boundaries. The permittivities of the materials used in the simulation (i.e., Ge, Cr, and Au) are provided in note S3.

### Sample fabrication

The details of the sample fabrication can be found in note S6.

### Optical characterization

The details of the experimental setup can be found in note S7.

### Data analysis

The details of post–data processing for SPP excitation and reflection can be found in notes S8 and S10, respectively.

## Supplementary Materials

### This PDF file includes:

Notes S1 to S10 Figs. S1 to S11 References

## References

[1] C. M. Bender, S. Boettcher, Real spectra in non-Hermitian Hamiltonians having PT symmetry. Phys. Rev. Lett. 80, 5243–5246 (1998).

[2] C. M. Bender, D. C. Brody, H. F. Jones, Must a Hamiltonian be Hermitian? Am. J. Phys. 71, 1095–1102 (2003).

[3] W. Wang, L. Q. Wang, R. D. Xue, H. L. Chen, R. P. Guo, Y. Liu, J. Chen, Unidirectional excitation of radiative-loss-free surface plasmon polaritons in PT-symmetric systems. Phys. Rev. Lett. 119, 077401 (2017). 28949654 10.1103/PhysRevLett.119.077401

[4] C. E. Rüter, K. G. Makris, R. el-Ganainy, D. N. Christodoulides, M. Segev, D. Kip, Observation of parity–time symmetry in optics. Nat. Phys. 6, 192–195 (2010).

[5] M. Lawrence, N. Xu, X. Zhang, L. Cong, J. Han, W. Zhang, S. Zhang, Manifestation of PT symmetry breaking in polarization space with terahertz metasurfaces. Phys. Rev. Lett. 113, 093901 (2014). 25215984 10.1103/PhysRevLett.113.093901

[6] M. C. Rechtsman, Optical sensing gets exceptional. Nature 548, 161–162 (2017). 28796210 10.1038/548161a

[7] W. Chen, S. Kaya Ozdemir, G. Zhao, J. Wiersig, L. Yang, Exceptional points enhance sensing in an optical microcavity. Nature 548, 192–196 (2017). 28796206 10.1038/nature23281

[8] H. Hodaei, A. U. Hassan, S. Wittek, H. Garcia-Gracia, R. el-Ganainy, D. N. Christodoulides, M. Khajavikhan, Enhanced sensitivity at higher-order exceptional points. Nature 548, 187–191 (2017). 28796201 10.1038/nature23280

[9] J. Wiersig, Enhancing the sensitivity of frequency and energy splitting detection by using exceptional points: Application to microcavity sensors for single-particle detection. Phys. Rev. Lett. 112, 203901 (2014).

[10] J.-H. Park, A. Ndao, W. Cai, L. Hsu, A. Kodigala, T. Lepetit, Y. H. Lo, B. Kanté, Symmetry-breaking-induced plasmonic exceptional points and nanoscale sensing. Nat. Phys. 16, 462–468 (2020).

[11] S. Assawaworrarit, X. Yu, S. Fan, Robust wireless power transfer using a nonlinear parity-time-symmetric circuit. Nature 546, 387–390 (2017). 28617463 10.1038/nature22404

[12] Y. Xu, J.-H. Jiang, H. Chen, Stable lossless polaritons on non-Hermitian optical interfaces. Phys. Rev. B 95, 041409(R) (2017).

[13] J. Luo, J. Li, Y. Lai, Electromagnetic impurity-immunity induced by parity-time symmetry. Phys. Rev. X 8, 031035 (2018).

[14] H. Zhao, W. S. Fegadolli, J. Yu, Z. Zhang, L. Ge, A. Scherer, L. Feng, Metawaveguide for asymmetric interferometric light-light switching. Phys. Rev. Lett. 117, 193901 (2016). 27858452 10.1103/PhysRevLett.117.193901

[15] L. Feng, Y. L. Xu, W. S. Fegadolli, M. H. Lu, J. E. B. Oliveira, V. R. Almeida, Y. F. Chen, A. Scherer, Experimental demonstration of a unidirectional reflectionless parity-time metamaterial at optical frequencies. Nat. Mater. 12, 108–113 (2013). 23178268 10.1038/nmat3495

[16] H. Alaeian, B. Baum, V. Jankovic, M. Lawrence, J. A. Dionne, Towards nanoscale multiplexing with parity-time-symmetric plasmonic coaxial waveguides. Phys. Rev. B 93, 205439 (2016).

[17] H. Alaeian, J. A. Dionne, Non-Hermitian nanophotonic and plasmonic waveguides. Phys. Rev. B 89, 205439 (2014).

[18] G. Harari, M. A. Bandres, Y. Lumer, M. C. Rechtsman, Y. D. Chong, M. Khajavikhan, D. N. Christodoulides, M. Segev, Topological insulator laser: Theory. Science 359, eaar4003 (2018). 29420260 10.1126/science.aar4003

[19] M. A. Bandres, S. Wittek, G. Harari, M. Parto, J. Ren, M. Segev, D. N. Christodoulides, M. Khajavikhan, Topological insulator laser: Experiments. Science 359, eaar4005 (2018). 29420263 10.1126/science.aar4005

[20] H. Zhao, X. Qiao, T. Wu, B. Midya, S. Longhi, L. Feng, Non-Hermitian topological light steering. Science 365, 1163–1166 (2019). 31515392 10.1126/science.aay1064

[21] B. Midya, H. Zhao, L. Feng, Non-Hermitian photonics promises exceptional topology of light. Nat. Commun. 9, 2674 (2018). 29991729 10.1038/s41467-018-05175-8 PMC6039517

[22] X. Ni, D. Smirnova, A. Poddubny, D. Leykam, Y. Chong, A. B. Khanikaev, PT phase transitions of edge states at PT symmetric interfaces in non-Hermitian topological insulators. Phys. Rev. B 98, 165129 (2018).

[23] B. Zhen, C. W. Hsu, Y. Igarashi, L. Lu, I. Kaminer, A. Pick, S. L. Chua, J. D. Joannopoulos, M. Soljǎić, Spawning rings of exceptional points out of Dirac cones. Nature 525, 354–358 (2015). 26352476 10.1038/nature14889

[24] Q. Song, M. Odeh, J. Zúñiga-Pérez, B. Kanté, P. Genevet, Plasmonic topological metasurface by encircling an exceptional point. Science 373, 1133–1137 (2021). 34516834 10.1126/science.abj3179

[25] A. Regensburger, C. Bersch, M. A. Miri, G. Onishchukov, D. N. Christodoulides, U. Peschel, Parity-time synthetic photonic lattices. Nature 488, 167–171 (2012). 22874962 10.1038/nature11298

[26] K. G. Makris, L. Ge, H. Türeci, Anomalous transient amplification of waves in non-normal photonic media. Phys. Rev. X 4, 041044 (2014).

[27] K. Makris, Transient growth and dissipative exceptional points. Phys. Rev. E 104, 054218 (2021). 34942815 10.1103/PhysRevE.104.054218

[28] H. Hodaei, M.-A. Miri, M. Heinrich, D. N. Christodoulides, M. Khajavikhan, Parity-time–symmetric microring lasers. Science 346, 975–978 (2014). 25414308 10.1126/science.1258480

[29] C. Hahn, S. H. Song, C. H. Oh, P. Berini, Single-mode lasers and parity-time symmetry broken gratings based on active dielectric-loaded long-range surface plasmon polariton waveguides. Opt. Express 23, 19922–19931 (2015). 26367652 10.1364/OE.23.019922

[30] B. Peng, Ş. K. Özdemir, S. Rotter, H. Yilmaz, M. Liertzer, F. Monifi, C. M. Bender, F. Nori, L. Yang, Loss-induced suppression and revival of lasing. Science 346, 328–332 (2014). 25324384 10.1126/science.1258004

[31] Y. D. Chong, L. Ge, A. D. Stone, PT-symmetry breaking and laser-absorber modes in optical scattering systems. Phys. Rev. Lett. 106, 093902 (2011). 21405622 10.1103/PhysRevLett.106.093902

[32] S. Longhi, PT-symmetric laser absorber. Phys. Rev. A 82, 031801 (2010).

[33] B. Baum, H. Alaeian, J. Dionne, A parity-time symmetric coherent plasmonic absorber-amplifier. J. Appl. Phys. 117, 063106 (2015).

[34] L. Feng, Z. J. Wong, R.-M. Ma, Y. Wang, X. Zhang, Single-mode laser by parity-time symmetry breaking. Science 346, 972–975 (2014). 25414307 10.1126/science.1258479

[35] A. Guo, G. J. Salamo, D. Duchesne, R. Morandotti, M. Volatier-Ravat, V. Aimez, G. A. Siviloglou, D. N. Christodoulides, Observation of PT-symmetry breaking in complex optical potentials. Phys. Rev. Lett. 103, 093902 (2009). 19792798 10.1103/PhysRevLett.103.093902

[36] S. Xiao, J. Gear, S. Rotter, J. Li, Effective PT-symmetric metasurfaces for subwavelength amplified sensing. New J. Phys. 18, 085004 (2016).

[37] N. Nye, A. Halawany, C. Markos, M. Khajavikhan, D. Christodoulides, Flexible PT-symmetric optical metasurfaces. Phys. Rev. Appl. 13, 064005 (2020).

[38] F. Yang, C. S. Prasad, W. Li, R. Lach, H. O. Everitt, G. V. Naik, Non-Hermitian metasurface with non-trivial topology. Nanophotonics 11, 1159–1165 (2022). 10.1515/nanoph-2021-0731 PMC11501583 39635073

[39] D. N. Basov, M. M. Fogler, F. J. Garcia de Abajo, Polaritons in van der Waals materials. Science 354, eaag1992 (2016). 10.1126/science.aag1992 27738142

[40] T. Low, A. Chaves, J. D. Caldwell, A. Kumar, N. X. Fang, P. Avouris, T. F. Heinz, F. Guinea, L. Martin-Moreno, F. Koppens, Polaritons in layered two-dimensional materials. Nat. Mater. 16, 182–194 (2017). 27893724 10.1038/nmat4792

[41] Z. Cai, Y. Xu, C. Wang, Y. Liu, Polariton photonics using structured metals and 2D materials. Adv. Opt. Mater. 8, 1901090 (2019).

[42] L. Li, T. Li, S. M. Wang, C. Zhang, S. N. Zhu, Plasmonic Airy beam generated by in-plane diffraction. Phys. Rev. Lett. 107, 126804 (2011). 22026786 10.1103/PhysRevLett.107.126804

[43] L. Li, T. Li, S. M. Wang, S. N. Zhu, Collimated plasmon beam: Nondiffracting versus linearly focused. Phys. Rev. Lett. 110, 046807 (2013). 25166192 10.1103/PhysRevLett.110.046807

[44] P. L. Stiles, J. A. Dieringer, N. C. Shah, R. P. Van Duyne, Surface-enhanced Raman spectroscopy. Annu. Rev. Anal. Chem. 1, 601–626 (2008). 10.1146/annurev.anchem.1.031207.112814 20636091

[45] J. N. Anker, W. P. Hall, O. Lyandres, N. C. Shah, J. Zhao, R. P. van Duyne, Biosensing with plasmonic nanosensors. Nat. Mater. 7, 442–453 (2008). 18497851 10.1038/nmat2162

[46] P. Berini, I. De Leon, Surface plasmon–polariton amplifiers and lasers. Nat. Photon. 6, 16–24 (2012).

[47] W. Srituravanich, L. Pan, Y. Wang, C. Sun, D. B. Bogy, X. Zhang, Flying plasmonic lens in the near field for high-speed nanolithography. Nat. Nanotechnol. 3, 733–737 (2008). 19057593 10.1038/nnano.2008.303

[48] M. L. Brongersma, V. M. Shalaev, The case for plasmonics. Science 328, 440–441 (2010). 20413483 10.1126/science.1186905

[49] T. W. Ebbesen, C. Genet, S. I. Bozhevolnyi, Surface-plasmon circuitry. Phys. Today 61, 44–50 (2008).

[50] J. Chen, Z. Li, S. Yue, Q. Gong, Efficient unidirectional generation of surface plasmon polaritons with asymmetric single-nanoslit. Appl. Phys. Lett. 97, 041113 (2010).

[51] B. Eftekharinia, A. Moshaii, A. Dabirian, Design of a slit-groove coupler for unidirectional excitation of the guided surface plasmon polaritons through a plasmonic slot waveguide. Plasmonics 12, 131–138 (2017).

[52] K. M. McPeak, S. V. Jayanti, S. J. P. Kress, S. Meyer, S. Iotti, A. Rossinelli, D. J. Norris, Plasmonic films can easily be better: Rules and recipes. ACS Photonics 2, 326–333 (2015). 25950012 10.1021/ph5004237 PMC4416469

[53] F. J. Rodríguez-Fortuño, G. Marino, P. Ginzburg, D. O’Connor, A. Martínez, G. A. Wurtz, A. V. Zayats, Near-field interference for the unidirectional excitation of electromagnetic guided modes. Science 340, 328–330 (2013). 23599487 10.1126/science.1233739

[54] J. Lin, J. P. B. Mueller, Q. Wang, G. Yuan, N. Antoniou, X. C. Yuan, F. Capasso, Polarization-controlled tunable directional coupling of surface plasmon polaritons. Science 340, 331–334 (2013). 23599488 10.1126/science.1233746

[55] Y. Liu, S. Palomba, Y. Park, T. Zentgraf, X. Yin, X. Zhang, Compact magnetic antennas for directional excitation of surface plasmons. Nano Lett. 12, 4853–4858 (2012). 22845720 10.1021/nl302339z

[56] J. Yang, S. Zhou, C. Hu, W. Zhang, X. Xiao, J. Zhang, Broadband spin-controlled surface plasmon polariton launching and radiation via L-shaped optical slot nanoantennas. Laser Photonics Rev. 8, 590–595 (2014).

[57] S. Sun, Q. He, S. Xiao, Q. Xu, X. Li, L. Zhou, Gradient-index meta-surfaces as a bridge linking propagating waves and surface waves. Nat. Mater. 11, 426–431 (2012). 22466746 10.1038/nmat3292

[58] H. Lingling, C. Xianzhong, B. Benfeng, T. Qiaofeng, J. Guofan, T. Zentgraf, S. Zhang, Helicity dependent directional surface plasmon polariton excitation using a metasurface with interfacial phase discontinuity. Light Sci. Appl. 2, e70 (2013).

[59] M. A. Bandres, M. Segev, Non-Hermitian topological systems. Phys. Ther. 11, 96 (2018).

[60] A. Hohenau, J. R. Krenn, A. Drezet, O. Mollet, S. Huant, C. Genet, B. Stein, T. W. Ebbesen, Surface plasmon leakage radiation microscopy at the diffraction limit. Opt. Express 19, 25749–25762 (2011). 22273967 10.1364/OE.19.025749

[61] X. Yang, J. Yao, J. Rho, X. Yin, X. Zhang, Experimental realization of three-dimensional indefinite cavities at the nanoscale with anomalous scaling laws. Nat. Photon. 6, 450–454 (2012).

[62] D. I. Yakubovsky, A. V. Arsenin, Y. V. Stebunov, D. Y. Fedyanin, V. S. Volkov, Optical constants and structural properties of thin gold films. Opt. Express 25, 25574–25587 (2017). 29041223 10.1364/OE.25.025574

[63] A. Ciesielski, L. Skowronski, W. Pacuski, T. Szoplik, Permittivity of Ge, Te and Se thin films in the 200–1500 nm spectral range. Predicting the segregation effects in silver. Mater. Sci. Semicond. Process. 81, 64–67 (2018).

[64] P. Johnson, R. Christy, Optical constants of transition metals: Ti, V, Cr, Mn, Fe, Co, Ni, and Pd. Phys. Rev. B 9, 5056–5070 (1974).

[65] G. Yoon, I. Kim, S. So, J. Mun, M. Kim, J. Rho, Fabrication of three-dimensional suspended, interlayered and hierarchical nanostructures by accuracy-improved electron beam lithography overlay. Sci. Rep. 7, 6668 (2017). 28751643 10.1038/s41598-017-06833-5 PMC5532261

