# Supplementary material for: Subwavelength control of light transport at the exceptional point by non-Hermitian metagratings
Source: Sci Adv. 2023 May 12;9(19):eadf3510. doi: 10.1126/sciadv.adf3510 (PMC10181182; doi:10.1126/sciadv.adf3510)
Supplement: Supplementary file 1 — Notes S1 to S10 Figs. S1 to S11 References [file sciadv.adf3510_sm.pdf]

Science Advances
AAAS

Supplementary Materials for

Subwavelength control of light transport at the exceptional point by non-Hermitian metagratings

Yihao Xu *et al.*

Corresponding author: Junsuk Rho, jsrho@postech.ac.kr; Yongmin Liu, y.liu@northeastern.edu

Sci. Adv. **9**, eadf3510 (2023)
DOI: 10.1126/sciadv.adf3510

This PDF file includes:

Notes S1 to S10
Figs. S1 to S11
References

1. Theoretical Analysis of Field Distribution of Excited SPPs

The permittivity perturbation modulated by the grating array can be expressed as

$$\begin{aligned}\varepsilon(x) &= \varepsilon_d + A[\cos(\beta x) - iV_0 \sin(\beta x - \phi)] + o(\cos 2\beta x, \sin 2\beta x)\\ &\equiv \varepsilon_d + A_L \exp(i\beta x) + A_R \exp(-i\beta x) + o(\exp(\pm 2i\beta x)) \qquad \text{(S1)}\end{aligned}$$

Here ε_d is the relative permittivity of the dielectric layer, $\beta \equiv k_{spp}$ is the wavenumber of excited SPPs at $\lambda_0 = 1150$ nm, ϕ represents additional phase shift for the imaginary modulation, and the rigorous PT symmetric condition only happens when $\phi = 0$ or $\phi = \pi$. The last term $o(\exp(\pm 2i\beta x))$ represents the high-order Fourier components (for a profile with strict PT symmetry, this term vanishes). $A_{R/L}$ can then be expressed as

$$A_R = A\left[\frac{1 + V_0 \exp(i\phi)}{2}\right], A_L = A\left[\frac{1 - V_0 \exp(-i\phi)}{2}\right] \qquad \text{(S2)}$$

The magnetic field of incident polarized light is given by

$$\vec{H_i} = \hat{y} H_0 \exp(ik_0 z) \qquad \text{(S3)}$$

Assuming r is the reflection coefficient, then the reflected wave is

$$\vec{H_r} = \hat{y} r H_0 \exp(-ik_0 z) \qquad \text{(S4)}$$

The excited SPP waves that propagate to the left and right can be expressed as

$$\begin{cases} \vec{H_L} = \hat{y} H_L \exp(i\beta x - \alpha z)\\ \vec{H_R} = \hat{y} H_R \exp(-i\beta x - \alpha z)\end{cases} \qquad \text{(S5)}$$

where $H_{L/R}$ represent the amplitude of the excited SPPs on the left/right side.

The total field is the combination of the four components:

$$\vec{H} = \vec{H_i} + \vec{H_r} + \vec{H_L} + \vec{H_R} \qquad \text{(S6)}$$

According to Maxwell's equation, $\nabla \times \vec{E} = -\mu_0 \frac{\partial \vec{H}}{\partial t} = -i\omega\mu_0 \vec{H}, \nabla \times \vec{H} = \varepsilon \frac{\partial \vec{E}}{\partial t} = i\omega\varepsilon\vec{E} + \sigma\vec{E}$.

When the perturbation is weak, $\varepsilon(x)$ can be approximated as a constant. Therefore, we have

$$\nabla \times \nabla \times \vec{H} = \nabla \times (i\omega\varepsilon_0\varepsilon(x) + \sigma)\vec{E} \cong (i\omega\varepsilon_0\varepsilon(x) + \sigma)\nabla \times \vec{E} = \left[\varepsilon(x)\frac{\omega^2}{c^2} - i\mu_0\sigma\omega\right]\vec{H} \qquad \text{(S7)}$$

where σ is the conductivity of the metal substrate. In our designed meta-gratings, along the horizontal direction (the propagation direction of SPPs), the filling ratios of the real part modulation and imaginary modulation are 0.07 and 0.14, respectively. Along the surface normal direction, compared to the penetration depth of SPPs in air ($d \sim 1.43\mu m$) at the working wavelength of 1150 nm, the ratios of the real part modulation and imaginary modulation are 0.056 and 0.036, respectively. Therefore, the modulation of the permittivity is indeed very weak. We further define $\tau = 2/(\mu_0\sigma c^2)$ and obtain

$$\nabla \times \nabla \times \vec{H} = \frac{1}{c^2}\left[\varepsilon(x)\omega^2 - \frac{2i}{\tau}\omega\right]\vec{H} \qquad \text{(S8)}$$

To derive the first term, we first calculate

$$\begin{aligned}\nabla \times \vec{H} &= \hat{z}\partial_x H_y - \hat{x}\partial_z H_y\\ &= i\beta\hat{z}\left[H_L e^{(i\beta x - \alpha z)} - H_R e^{(-i\beta x - \alpha z)}\right] + \alpha\hat{x}\left[H_L e^{(i\beta x - \alpha z)} + H_R e^{(-i\beta x - \alpha z)}\right]\\ &\quad - ik_0\hat{x}H_0\left(e^{ik_0 z} - re^{-ik_0 z}\right) \qquad \text{(S9)}\end{aligned}$$

And therefore

$$\nabla \times \nabla \times \vec{H} = \hat{y}\left[(-\alpha^2 + \beta^2)\left(H_L e^{i\beta x - \alpha z} + H_R e^{-i\beta x - \alpha z}\right) + k_0^2 H_0\left(e^{ik_0 z} + re^{-ik_0 z}\right)\right] \qquad \text{(S10)}$$

We know that $\beta^2 - \alpha^2 = k_0^2$, and let $\varphi(z) \equiv \exp(ik_0 z) + r\exp(-ik_0 z)$. Then we have

$$\nabla \times \nabla \times \vec{H} = \hat{y}k_0^2[(H_L \exp(i\beta x) + H_R \exp(-i\beta x))e^{-\alpha z} + H_0\varphi(z)] \qquad \text{(S11)}$$

Combine Equations (S3-S8) and (S11), we obtain

$$[1 + A_R \exp(-i\beta x) + A_L \exp(i\beta x)]\omega^2[H_0\varphi(z) + (H_L \exp(i\beta x) + H_R \exp(-i\beta x))e^{-\alpha z}]$$
$$= \left(\omega_0^2 + i\frac{2}{\tau}\omega\right)[H_0\varphi(z) + (H_L \exp(i\beta x) + H_R \exp(-i\beta x))e^{-\alpha z}] \quad \text{(S12)}$$

Here, $\omega_0 = ck_0$ represents to frequency of free-space light. Multiply $\varphi^*(z)$ on both sides and do the integration from $z = 0$ to $z = d$, where d is the penetration depth of SPPs in air along z axis. If we define $N \equiv \int_0^d \varphi(z)\varphi^*(z)dz$, and $V \equiv \int_0^d e^{-\alpha z}\varphi^*(z)dz$, we obtain

$$[1 + A_R \exp(-i\beta x) + A_L \exp(i\beta x)]\omega^2[H_0NV^{-1} + H_L \exp(i\beta x) + H_R \exp(-i\beta x)]$$
$$= (\omega_0^2 + i\frac{2}{\tau}\omega)[H_0NV^{-1} + H_L \exp(i\beta x) + H_R \exp(-i\beta x)] \quad \text{(S13)}$$

The coefficient for $\exp(in\beta x), n = \pm 1,0$ should match and we can rewrite the equation as

$$\begin{cases} \left(\omega_0^2 - \omega^2 + i\frac{2}{\tau}\omega\right)\begin{pmatrix} H_L \\ H_R \end{pmatrix} = \omega^2 NV^{-1} \cdot H_0 \begin{pmatrix} A_L \\ A_R \end{pmatrix}, n = \pm 1 \\ \left(\omega_0^2 - \omega^2 + i\frac{2}{\tau}\omega\right)NV^{-1} \cdot H_0 = \omega^2(A_RH_L + A_LH_R), n = 0 \end{cases} \quad \text{(S14)}$$

Notice $\omega \approx \omega_0 \Rightarrow (\omega_0^2 - \omega^2) \approx 2\omega(\omega_0 - \omega)$ under the weak perturbation, we can get

$$\begin{cases} \left(\omega_0 - \omega + \frac{i}{\tau}\right)\begin{pmatrix} H_L \\ H_R \end{pmatrix} = \frac{\omega}{2}NV^{-1}H_0 \begin{pmatrix} A_L \\ A_R \end{pmatrix} \\ \left(\omega_0 - \omega + \frac{i}{\tau}\right)NV^{-1}H_0 = \frac{\omega}{2}(A_RH_L + A_LH_R) \end{cases} \quad \text{(S15)}$$

The first equation in Equation (S15) is an equivalent form to the equations under coupled mode theory(*61*) when considering $i\omega \to d/dt$ and defining $\sqrt{\frac{1}{\tau_{L,R}}} = -\frac{i\omega}{2}NV^{-1}A_{L,R}$:

$$\begin{cases} \frac{dH_L}{dt} = \left(i\omega_0 - \frac{1}{\tau}\right)H_L + \sqrt{\frac{1}{\tau_L}} \cdot H_0 \\ \frac{dH_R}{dt} = \left(i\omega_0 - \frac{1}{\tau}\right)H_R + \sqrt{\frac{1}{\tau_R}} \cdot H_0 \end{cases} \quad \text{(S16)}$$

From the first equation in Equation (S15), and define $\tilde{\omega}_0 \equiv \omega_0 + i/\tau$, we can get

$$H_L = \frac{\omega NV^{-1}H_0}{2(\tilde{\omega}_0 - \omega)} \cdot A_L, \qquad H_R = \frac{\omega NV^{-1}H_0}{2(\tilde{\omega}_0 - \omega)} \cdot A_R \quad \text{(S17)}$$

Dividing the two equations gives rise to

$$\left|\frac{H_R}{H_L}\right| = \left|\frac{A_R}{A_L}\right| = \left|\frac{1 + V_0 \exp(i\phi)}{1 - V_0 \exp(-i\phi)}\right| \quad \text{(S18)}$$

Therefore, we conclude that the amplitude of excited SPPs in both sides are proportional to the 1st order Fourier coefficient of the modulated permittivity. Furthermore, we emphasize that the unidirectional excitation of SPPs ($A_L = 0$ or $A_R = 0$) only happens when $(V_0, \phi) = (1,0)$ or $(V_0, \phi) = (1, \pi)$, corresponding to the EPs in *PT* symmetric cases.

2. The Exceptional Point (EP) Behavior

To solve the eigenfrequencies of the modes in this system, we combine the two equations in Equation (S15) and notice $H_RA_L = H_LA_R$ from Equation (S17), we then get,

$$(\omega - \tilde{\omega}_0)^2 \begin{pmatrix} H_L \\ H_R \end{pmatrix} = \frac{\omega^2}{2}A_LA_R \begin{pmatrix} H_L \\ H_R \end{pmatrix} \quad \text{(S19)}$$

Since $A_L A_R \sim A^2 \ll 1$, the eigenvalues of the system can be obtained, which are given by

$$\lambda_{\pm} \equiv \frac{\omega_{\pm}}{\widetilde{\omega}_0} \cong 1 \pm \sqrt{\frac{A_L A_R}{2}} = 1 \pm \Delta\lambda\sqrt{1 - V_0^2 + 2iV_0 \sin\phi} \qquad \text{(S20)}$$

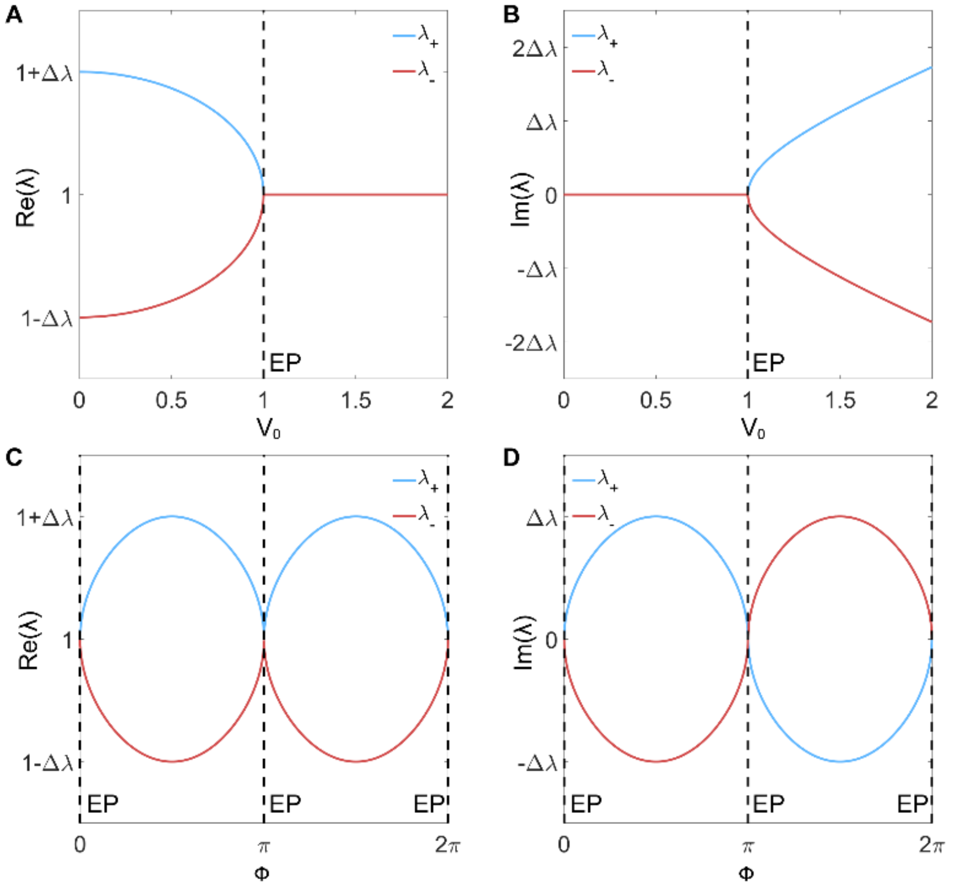

Fig. S1. Eigenvalue evolution of the non-Hermitian system. (**A**) The real part and (**B**) imaginary part of the eigenvalues with the respect to V_0, when ϕ is set to be 0. (**C**) The real part and (**D**) imaginary part of the eigenvalues with the respect to ϕ, when V_0 is set to be 1.

where $\Delta\lambda \equiv \frac{A}{2\sqrt{2}}$, and A, V_0, ϕ follow the definition in Equation (S1). The evolution of the eigenvalues with respect to the individual parameter (perturbation strength of the imaginary modulation V_0 and additional phase shift of the imaginary modulation ϕ) is shown in Fig. S1. Only $V_0 \geq 0$ regions are plotted in Fig. S1 (A and B), because the plot is symmetric with respect to $V_0 = 0$. Since λ_0 is a constant value, it is not plotted in Fig. 2 (A and B) in the main text to make the figure concise.

From Equation (S20) and also the eigenvalues plot, we can readily find that the bifurcation occurring at $1 - V_0^2 + 2iV_0 \sin\phi = 0 \Rightarrow (V_0, \phi) = (1,0)$ and $(V_0, \phi) = (1, \pi)$. In other words, the condition that $(V_0, \phi) = (1,0)$ and $(V_0, \phi) = (1, \pi)$ correspond to the EPs of this non-Hermitian system. From Equation (S2) and (18), this condition is equivalent to $A_L = 0$ or $A_R = 0$, leading to the ideal directional excitation of SPPs at the EP.

3. Design of non-Hermitian Meta-gratings for Unidirectional Excitations of SPPs

As shown in Fig. S2, the nano-strips are placed at the peak position of ε_r ($\propto \cos\beta x$) and ε_i

($\propto \sin\beta x$). Based on Fourier expansion, the permittivity profile can be decomposed into the linear combination of exponential functions:

$$\varepsilon(x) = \varepsilon_{air} + A_L \exp(i\beta x) + A_R \exp(-i\beta x) + \sum_{n=2}^{\infty}[A_{nL} \exp(in\beta x) + A_{nR} \exp(-in\beta x)] \quad \text{(S21)}$$

The geometric parameters of the strips are elaborately designed, so that $A_R \gg A_L$ and the high-order Fourier components can be ignored. The nano-strips are made of Germanium (Ge) and Chromium (Cr) on a 60 nm-thick gold (Au)-deposited silica substrate (*62*). In one unit cell, the nano-strip on the left is made of only Ge to modulate the real part of the permittivity profile thanks to the high index of Ge (*63*); the nano-strip on the right consists of both Ge and Cr layers, which perform as imaginary modulation due to the large imaginary permittivity of Cr (*64*).

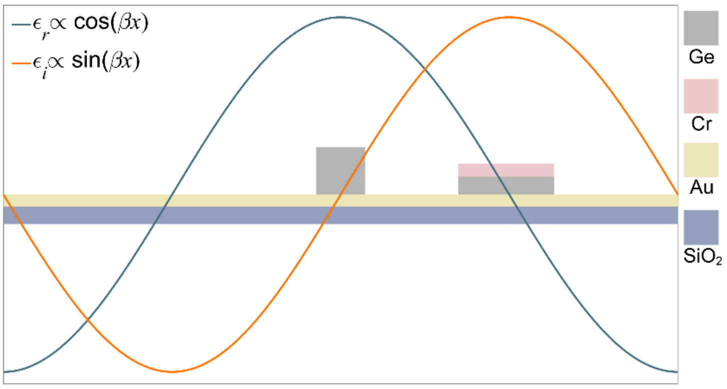

Fig. S2. Illustration of meta-gratings to approximate the PT-symmetric permittivity modulation.

The permittivity profile in one period of the nano-strip grating can be expressed as

$$\varepsilon(x) = 1 + A\left[\left(\varepsilon_{nb}^{(1)} - 1\right) h_{nb}^{(1)} \cdot rect(x, w_1) + \left(\varepsilon_{nb}^{(2)} - 1\right) h_{nb}^{(2)} \cdot rect(x - s, w_2)\right] \quad \text{(S22)}$$

where $\varepsilon_{nb}^{(i)}$ and $h_{nb}^{(i)}$ are the effective permittivity and height of nano-strip i $(i = 1, 2)$. A is the perturbation strength which will not affect the contrast. $rect(x, w)$ is a piecewise function:

$$rect(x, w) = \begin{cases} 1, & \left(-\frac{w}{2} \le x \le \frac{w}{2}\right) \\ 0, & \text{(otherwise)} \end{cases} \quad \text{(S23)}$$

Comparing Equation (S21) and (S22), we can calculate the nth Fourier components of the profile by

$$A_{nL} = \frac{1}{\Lambda}\int_{-\Lambda/2}^{\Lambda/2} \varepsilon(x) \exp(-in\beta x)\, dx \quad \text{(S24)}$$

$$A_{nR} = \frac{1}{\Lambda}\int_{-\Lambda/2}^{\Lambda/2} \varepsilon(x) \exp(in\beta x)\, dx \quad \text{(S25)}$$

Substituting $w_1 = 83$ nm, $w_2 = 162$ nm, $s = 280$ nm, and $n = 1$, into Equation (S24) and (S25), we obtain

$$\begin{cases} A_R = 0.1011A \\ A_L = 0.00917A \end{cases} \Rightarrow \frac{A_R}{A_L} > 10 \quad \text{(S26)}$$

Therefore, the optimized geometric parameters ensure that $A_R \gg A_L$

4. Simulation of Hermitian Nano-gratings

To verify the necessity and significance of the imaginary modulation with Ge/Cr, we perform an additional set of simulations for a Hermitian system without imaginary modulation. In these simulations, we substitute all the Cr to Ge, and therefore the two gratings are both made of pure Ge, as illustrated in Fig. S3A. Then, we sweep the geometric parameters to find an optimized design that shows the highest excitation contrast C_{exc} defined in the main text. The parameters include the heights of the two nano-strips (h_1 and h_2, ranging from 40 nm to 100 nm), and the widths of two nano-strips (w_1 and w_2, ranging from 80 nm to 180 nm). Each parameter is swept with 10 nm in step, resulting in ~6000 combinations in total. The histogram of the field ratio $|H_R/H_L|$ for all combinations are plotted in Fig. S3B. We find that the geometry of best design is $h_1 = h_2 = 100\ \mathrm{nm}$, $w_1 = 110\ \mathrm{nm}$ and $w_2 = 170\ \mathrm{nm}$, achieving field ratio $|H_R/H_L|$ around 3. In sharp contrast, the field ratio of the optimized non-Hermitian meta-grating can reach around 100 (red dashed line in Fig. S3B). It is worth pointing out that the real modulation system is only a reasonable approximation of a Hermitian system rather than an ideal Hermitian system, since the scattering loss of the two nano-strips is still different. Therefore, the excitation contrast will still be non-zero in these Hermitian systems, as proved in the next paragraph.

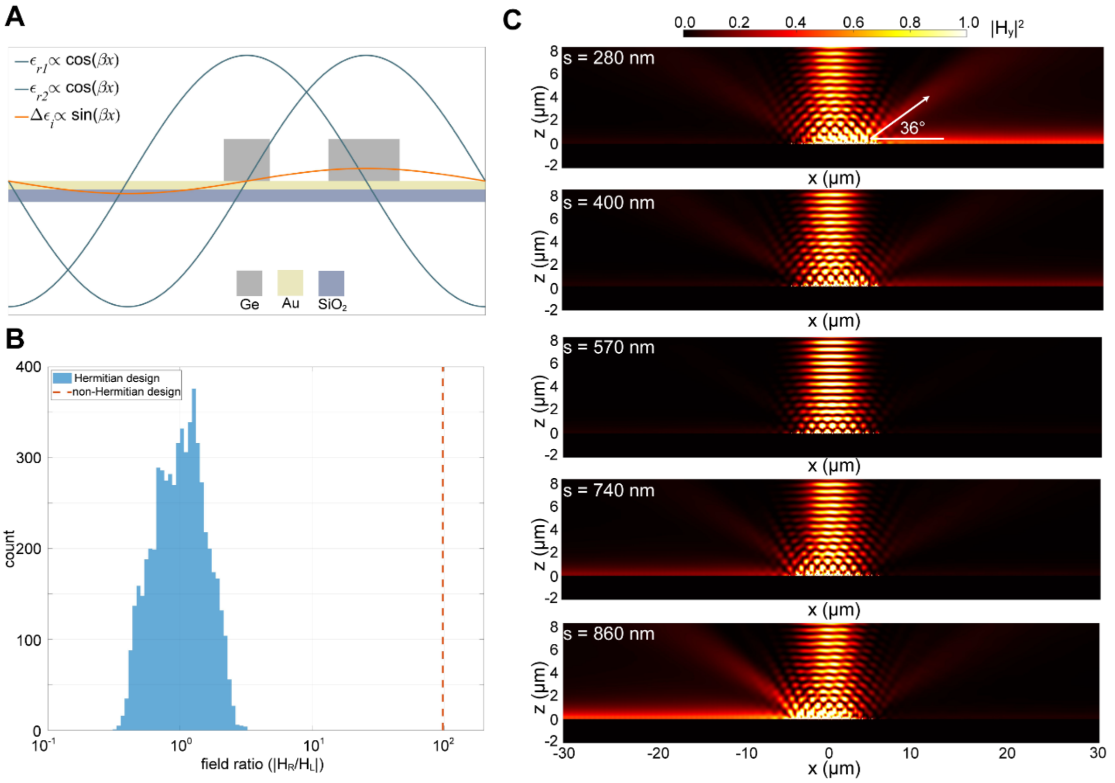

Fig. S3. Numerical simulation of SPP excitation in Hermitian systems. (**A**) Illustration of Hermitian gratings made of pure Ge with only real permittivity modulation. (**B**) Histogram of the field ratio ($|H_r/H_l|$) of all parameter combinations in the optimization process for the Hermitian grating design. The red dashed line is the field ratio of the best non-Hermitian meta-grating design presented in the main text. (**C**) Simulated magnetic field intensity for a Hermitian system when the separation distance of the two nano-strips in the unit cell is $s = 280\ \mathrm{nm}$, $400\ \mathrm{nm}$, $570\ \mathrm{nm}$, $740\ \mathrm{nm}$, $860\ \mathrm{nm}$.

For the optimal Hermitian design, the shift s is swept from 280 nm to 860 nm, and the field distributions are depicted in Fig. S3C. When we compare Fig. S3C (the optimized Hermitian system with the highest excitation contrast) to Fig. 2 (the optimized non-Hermitian system), we see that the excitation of SPPs in a Hermitian system, even after optimization, cannot achieve the same level of contrast as the non-Hermitian system, although asymmetric excitation of SPPs still exists. This non-zero field contrast is owing to the asymmetric arrangement of the two Ge nano-strips, which results in the interference between the excitation from the two Ge nano-strip. The higher order Fourier components also contribute to this asymmetric field excitation. It should also be noted that the two nano-strips have a modest imaginary permittivity difference which, as previously stated, is not an ideal Hermitian system due to the scattering loss. Furthermore, adopting two pure Ge gratings in one unit cell has two additional drawbacks: On the one hand, the contrast of non-Hermitian meta-gratings can be easily controlled by adjusting the shift s. In the meantime, this shift control has a low impact on excitation efficiency, as can be observed in Fig. 2. However, once the shift s approaches half of the period ($\Lambda/2 = 570\,\mathrm{nm}$), the best Hermitian design has a dramatically lower excitation efficiency. The Hermitian grating at $s = \Lambda/4$, on the other hand, exhibits substantial diffraction in the $36°$ direction, as illustrated in Fig. S3C. Both phenomena are attributed to the similar scattering phase of the two Ge nano-strips. If we consider the extreme scenario where the two gratings are identical, even though the period of the unit cell is Λ, we will get another effective period of $\Lambda + s$. As a result, secondary diffraction with a smaller wavenumber $2\pi/(\Lambda + s)$ will be generated. This secondary diffraction corresponds to a $35.9°$ diffraction angle when $s = \Lambda/4$, in good agreement with the simulation result. Moreover, when $s = \Lambda/2$, all gratings have the same distance between them. Consequently, the main period of the grating array will decrease to $\Lambda/2$, which corresponds to a larger wavenumber provided by the grating. The momentum mismatch cannot be rectified in this case, resulting in substantially low excitation efficiency of SPPs. Despite the fact that the two gratings in the simulation are not identical, the very similar scattering phase will still play a role in the two behaviors. In a word, we conclude that the imaginary modulation with Ge/Cr is critical for the realization of EPs in the non-Hermitian plasmonic system, as well as for the controllable SPP excitation with low back scattering.

5. Topological Properties at EP and Robustness Analysis of the Unidirectionality

To study the topological properties in the vicinity of the EPs, we first define a complex function of the parameter $R = (V_0, \phi)$ as

$$F(R) = A_R \cdot A_L \propto 1 - V_0^2 + 2iV_0 \sin\phi \tag{S27}$$

The benefits of this parameter definition are: (1) The zero points of $F(R)$ only occur at the EPs where $A_R = 0$ or $A_L = 0$. (2) $F(R)$ is positively related to the amplitude of excited SPPs in both directions (i.e., H_R and H_L), as proved in Note 1 in the Supplementary Materials. Then, we select an arbitrary closed loop Γ in the parameter space, and calculate the phase accumulation of $F(R)$ and the winding number w that is defined as (*24*)

$$w = \frac{1}{2\pi}\oint d\phi_F = \frac{1}{2\pi}\oint_\Gamma (\nabla \arg(F)) \cdot d\boldsymbol{l} \tag{S28}$$

There are four scenarios in the selection of loop Γ: (i) the loop encircles zero EPs, (ii) the loop encircles the EP on the left, (iii) the loop encircles the EP on the right, and (iv) the loop encircles both EPs. Examples of each scenario are plotted in Fig. S4, including the trajectories of R in the

parameter space and the function $F(R)$.

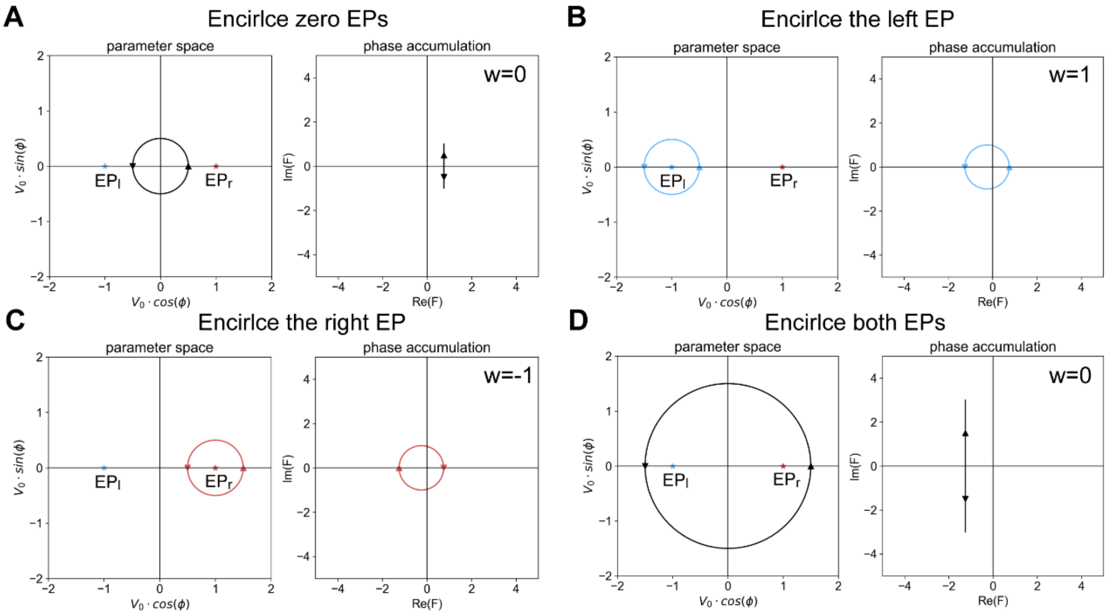

Fig. S4. The closed loop in the parameter space (left panels) and the corresponding phase accumulation (right panels) of F(R) for four scenarios. The loop encircles (**A**) zero EPs, (**B**) the left EP, (**C**) the right EP, (**D**) both EPs. The arrows in the left and right panels indicate the parameter sweeping direction and phase accumulation direction, respectively. The winding number w is also shown in each scenario.

As shown in Fig. S4, when the loop does not encircle zero EPs, a trivial topology exhibits and the winding number is zero. Interestingly, when the loop encircles the left (right) EP, non-trivial topology emerges, showing a non-zero winding number to be 1 (-1). The opposite winding number indicates that the two EPs have opposite topological charges. Finally, when the loop encircles both EPs, the total topological charge number is zero, and the system returns to a trivial topology with a zero winding number.

Then, we prove that the non-Hermitian system is more robust at the non-trivial topological singularities than the optimized Hermitian system in terms of maintaining high field contrast under perturbation. Without losing generality, we assume that the permittivity is given by

$$\varepsilon(x) = \varepsilon_d + A[\cos(\beta x) + (V_r - iV_0)\sin(\beta x - \phi)] \tag{S29}$$

For the PT-symmetric non-Hermitian system we considered, we have $V_r = 0$, meaning the imaginary part of the second nanostructures in the unit cell dominates. For a Hermitian system, we have $V_r \gg V_0 > 0$, where the real part of the second nanostructures in the unit cell dominates. The variable V_0, in this case, represents the difference in the imaginary part (including scattering loss) of the two real gratings as described in the previous note. Similar to Equation (S18), now we have

$$\left|\frac{H_R}{H_L}\right| = \left|\frac{A_R}{A_L}\right| = \left|\frac{1 + (V_0 + iV_r)\exp(i\phi)}{1 - (V_0 + iV_r)\exp(-i\phi)}\right| \tag{S30}$$

Therefore, the excitation contrast can be defined as:

$$C_{\text{exc}} = \frac{|H_R|^2 - |H_L|^2}{|H_R|^2 + |H_L|^2} = \frac{2V_0\cos\phi}{1 + V_0^2 + V_r^2 - 2V_r\sin\phi} \tag{S31}$$

By applying the requirements for V_0 and V_r to the non-Hermitian and Hermitian systems with specified shift (i.e., $\phi = \pi/2$), we have

$$C_{\text{exc}} = \begin{cases} \dfrac{2V_0}{1+V_0^2}, & (\text{non}-\text{Hermitian}) \\ \dfrac{2V_0}{1+V_r^2}, & (\text{Hermitian}) \end{cases} \tag{S32}$$

Then, to study the robustness of the contrast, we apply differentiation to both formula and obtain

$$\Delta C_{\text{exc}} = \begin{cases} \dfrac{2(1-V_0)^2}{(1+V_0^2)^2}\Delta V_0, & (\text{non}-\text{Hermitian}) \\ -\dfrac{4V_rV_0}{(1+V_r^2)^2}\Delta V_r + \dfrac{2}{1+V_r^2}\Delta V_0, & (\text{Hermitian}) \end{cases} \tag{S33}$$

When the perturbation is present in the non-Hermitian system at the EPs, that is, $V_0 = 1$, we get $\Delta C_{\text{exc}} = 0$, meaning that the excitation contrast is stable. However, ΔC_{exc} for a Hermitian system is non-zero regardless of whether V_r or V_0 is perturbed. Although it is still possible to have $\Delta C_{\text{exc}} = 0$ by precisely regulating the perturbations ΔV_r and ΔV_0 according to the equation, this scenario is beyond our discussion scope since we will focus on random perturbations (such as fabrication imperfection). As a result, we can theoretically confirm that the non-Hermitian system is robust to a permittivity perturbation when it is at the EPs.

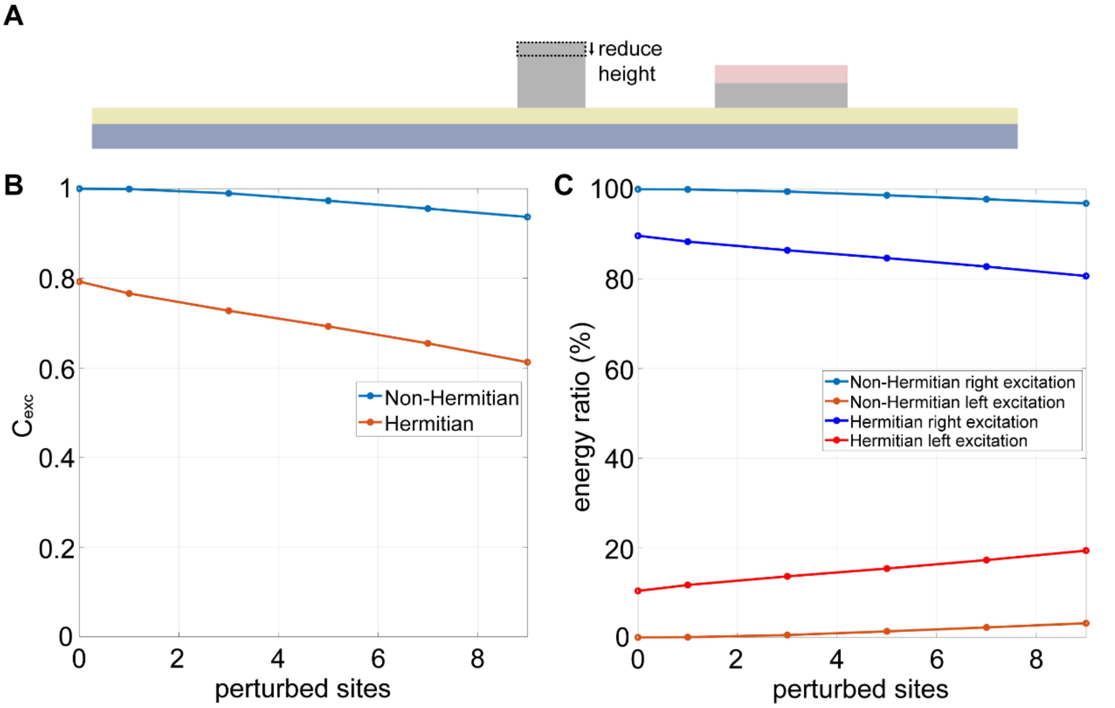

Fig. S5. Robustness analysis of the Hermitian and non-Hermitian systems. (**A**) Illustration of adding the perturbation to both non-Hermitian and Hermitian systems by reducing the height of the first grating in the unit cells. (**B**) Excitation contrast when the perturbation is added to $n \in [0, 9]$ unit cells in the non-Hermitian (light blue line) and Hermitian (orange line) systems. (**C**) Excitation energy ratio $I_{R/L}/(I_R + I_L)$ when the perturbation is added to $n \in [0, 9]$ unit cells in the non-Hermitian (light blue/orange line for right/left excitation) and Hermitian (blue/red line for right/left excitation) systems.

We then utilize full-wave simulations to further demonstrate the robustness of the non-Hermitian system. In both Hermitian and non-Hermitian systems, we reduce the height of the first grating by 20 nm for n ($n \in [0,9]$) unit cells among the 9 unit cells in each simulation, as shown in Fig. S5A. Then, we detect the excitation contrast C_{exc} as well as the excited energy ratio of SPP on both sides, as a function of the perturbed site number. The results are depicted in Fig. S5 (B and C). The excitation contrast in the non-Hermitian system is robust (always >0.93) as the number of perturbed sites grows, as shown in Fig. S5B. It is noted that the contrast does not alter at all as the number of disturbed sites increases from 0 to 1. In the Hermitian system, on the other hand, the excitation contrast lowers from 0.8 to 0.6. which is around three-fold decrease compared to the non-Hermitian system. The energy ratio $I_{R/L}/(I_R + I_L)$ of the excited SPP in both directions (Fig. S5C) shows a similar behavior. As the number of perturbed sites increases in the non-Hermitian system, we can clearly see that the energy on the right side is always around unity, whereas the energy on the left side is always around zero. These results strongly support our theoretical conclusion that the non-Hermitian meta-grating is robust under permittivity perturbation and can maintain unidirectional excitation at the EPs.

6. Sample Fabrication

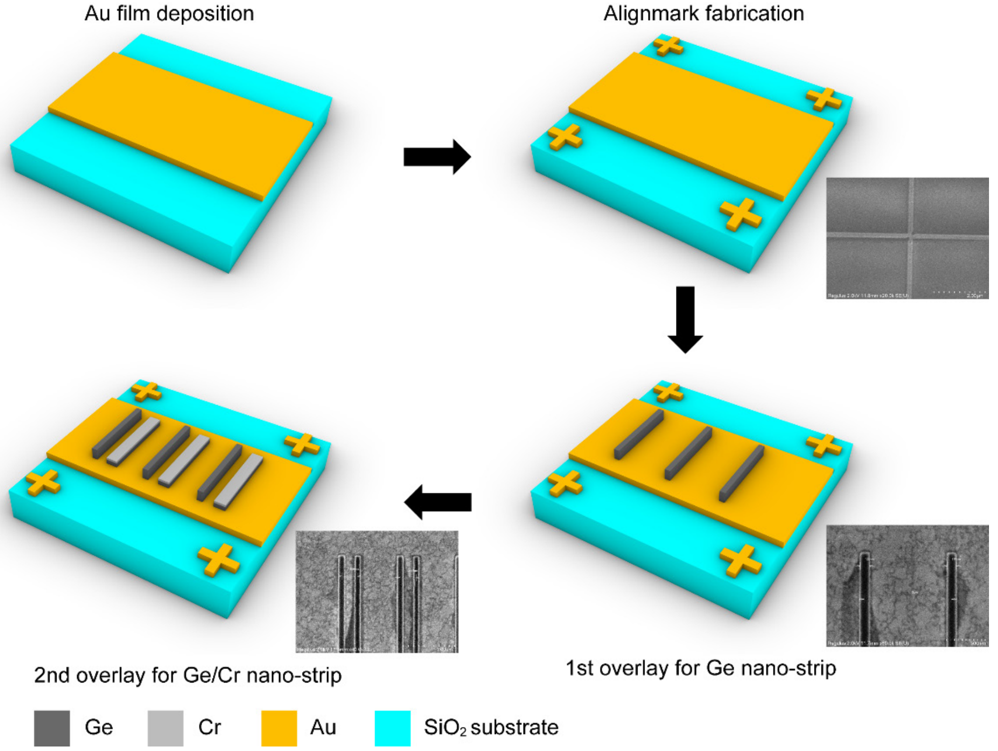

Fig. S6. Fabrication process and SEM images of the non-Hermitian meta-grating sample for directional excitation of SPPs.

The scheme of the whole fabrication process is shown in Fig. S6. The grating sample fabrication starts on a 150 μm silica substrate where 60 nm-thick Au film is deposited by electron-beam evaporation (KVT, KVE-E4000). An Au film with 3 nm Cr adhesion is deposited except the position where an alignment mark is to be made. On the substrate, copolymer (Microchem MMA (8.5) MAA EL8, 5000 rpm for 60 seconds, 160 °C bake for 5 minutes) and polymethyl

methacrylate (PMMA) (Microchem, 495 PMMA A2, 2000 rpm for 60 seconds, 180 °C bake for 5 minutes) are spin-coated. To reduce the charging effect by the dielectric, we spin-coat a conductive polymer layer (Showa Denko, E-spacer 300Z, 2000 rpm for 60 seconds). The alignment mark pattern is exposed by standard electron beam lithography (EBL) (Elionix, ELS-7800, 80 kV, 50 pA). After exposure, the conductive layer is washed with de-ionized (DI) water, and the bilayer resist is developed in the MIBK: IPA 1:3 solutions for 10 minutes at 4 °C. Then, 50 nm Au film with 3 nm Cr adhesion is deposited by electron-beam evaporation, and lift-off process is carried out in hot acetone at 50 °C. Using this standard EBL process, the Au alignment mark for the subsequent overlay process is fabricated. Next, the first overlay fabrication of 80 nm-thick Ge nano-strip with 3 nm Cr adhesion proceed by EBL for excitation grating. Before exposure of the electron beam, the alignment error in the overlay process is corrected with the Au alignment mark(*65*). Finally, the second overlay fabrication of 30 nm-thick Ge and 22 nm-thick Cr nano-strip with 3 nm Cr adhesion is proceeded by the standard EBL for the excitation grating. For the reflective meta-grating structure, it is made by 48 nm-thick Ge nano-strip with 3 nm Cr adhesion in the first overlay process, and 16 nm-thick Ge and 26 nm-thick Cr nano-strip with 3 nm Cr adhesion in the second overlay process. The two nano-strips with different heights and materials should have a high level of alignment, so a high-accuracy EBL overlay is required.

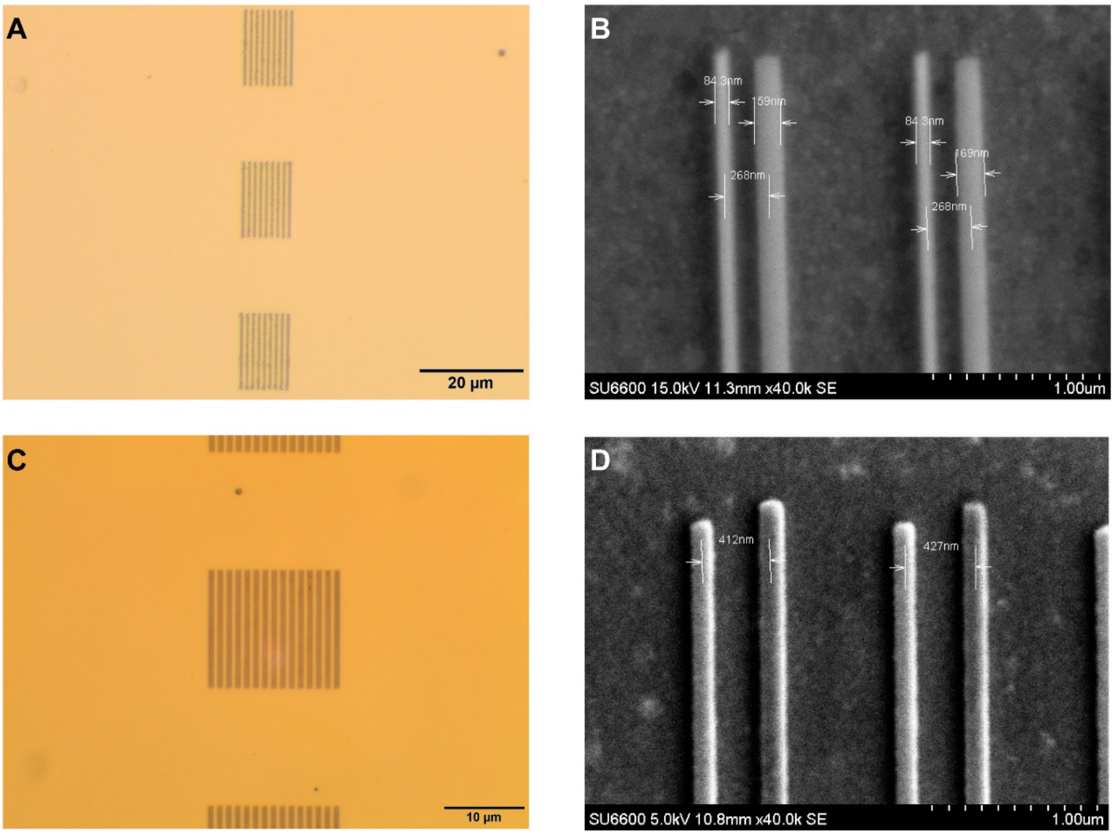

Fig. S7. Characterization of the fabricated samples. (**A**) Optical microscope image and (**B**) SEM image of the non-Hermitian meta-grating sample for directional excitation of SPPs. (**C**) Optical microscope image and (**D**) SEM image of the non-Hermitian meta-grating for directional reflection of SPPs.

The microscope image and SEM image of the fabricated sample for directional excitation of SPPs are shown in Fig. S7 (A and B). In this sample, the designed width of the gratings in one

unit cell is 83 nm and 162 nm, respectively. From Fig. S7B, we can see that the fabrication only had an error of less than 10 nm. The optical microscope and SEM images of the fabricated sample for directional reflection of SPPs are also captured, which are shown in Fig. S7 (C and D), respectively.

7. Experimental Setup

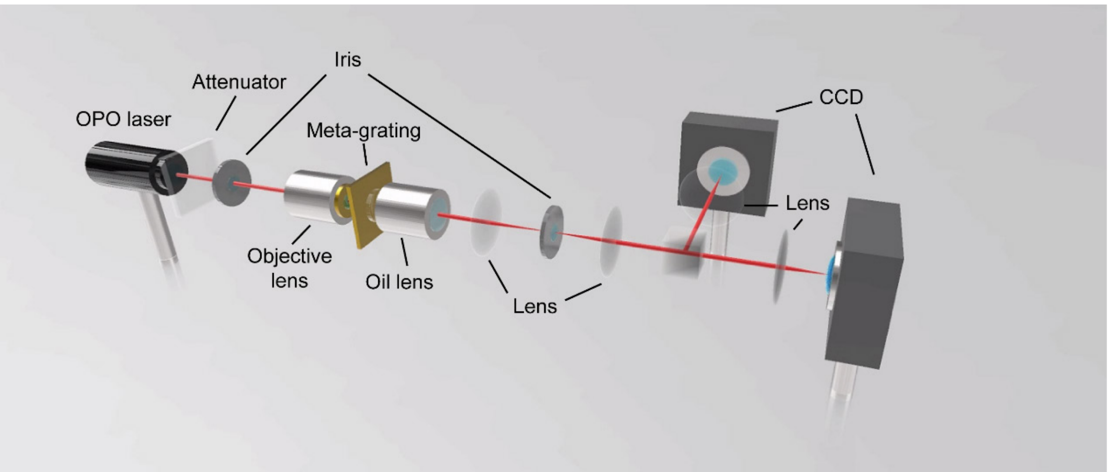

Fig. S8. Schematic of the experimental setup to image SPPs in real space and Fourier space.

The experimental setup to characterize SPPs measurement is shown in Fig. S8. An optical parametric oscillator (OPO) system (Chameleon Compact OPO VIS System) pumped by a Ti: Sapphire laser is used to generate the horizontally polarized laser beam at $\lambda = 1150$ nm (with a direct laser output power of 350mW). The laser beam first passes through an attenuator and an iris to get proper amplitude and beam spot. Then the beam is focused by an objective lens (Olympus 10×, with NA to be 0.25) on the sample to excite SPPs. The propagating SPP waves are coupled out to the free space by an immersed oil lens (Olympus PlanApo 60×, with NA to be 1.40). The gap between the oil lens and the backside of the sample is filled with immersion oil (refractive index $n = 1.52$). The light then passes through a 4-f system (two lenses and an iris) to eliminate the environmental noise. Finally, the light is split into two pathways by a beam splitter, and the real space and Fourier space images are captured by two charge-coupled devices (CCDs, Infinity 1-2CB), respectively.

8. Calculation of the Field Contrast of Excited SPPs

The method to extract and compare the field intensity of the generated SPPs is illustrated in Fig. S9. In the measurement, we first capture real space images (e.g., Fig. S9A) and Fourier space images (e.g., Fig. S9B). The Fourier space image essentially condenses the intensity of the left/right propagating SPPs to the two discrete arcs in the Fourier space. We then convert the Fourier image to a gray image as shown in Fig. S9C. Since the pixel value in the CCD image is proportional to total photons that reach the CCD in a given time, we can calculate the relative intensity of the SPPs in both directions by summing the pixel value of the two regions ($\pm k_{spp}$). In the example shown in Fig. S9C, the intensity ratio between the left/right propagating SPPs is $12571{:}9908 = 1.27$.

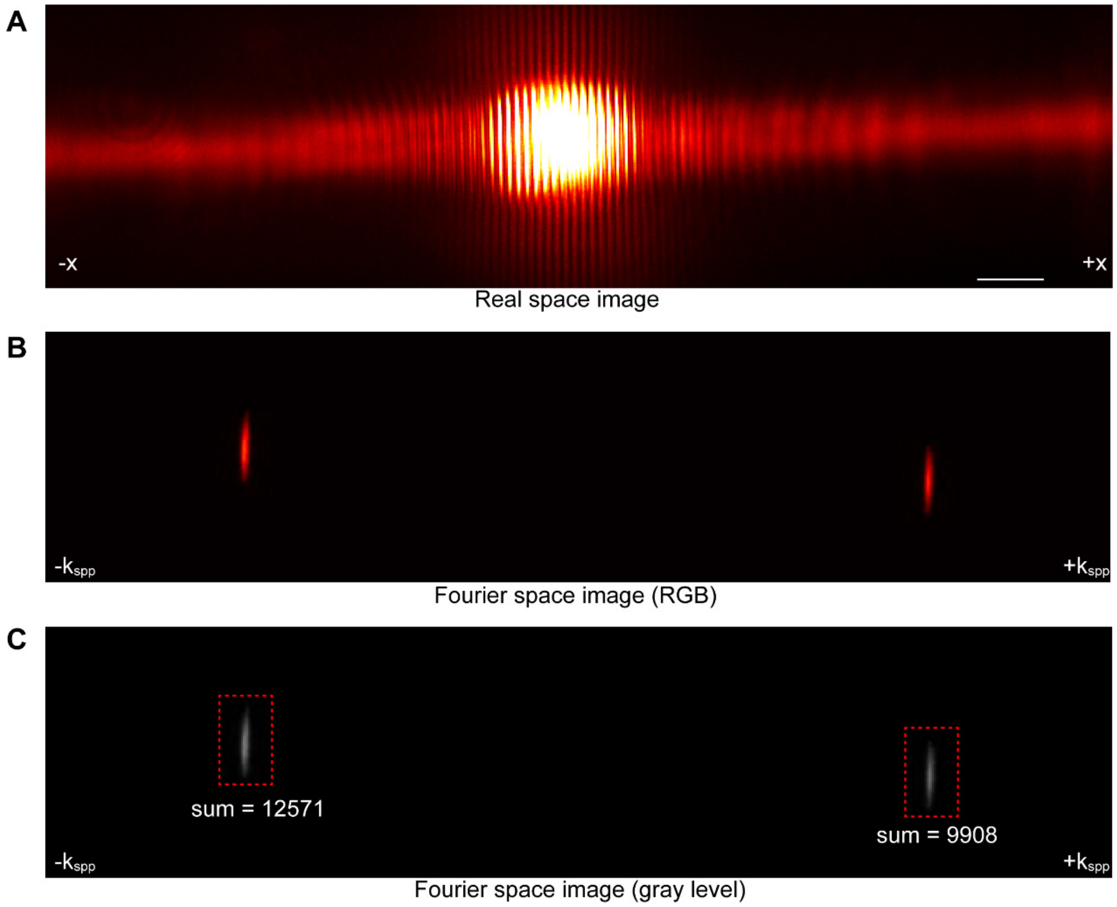

Fig. S9. Method to extract the field contrast of excited SPPs. (**A**) Example of real-space image of excited SPPs from a non-Hermitian meta-grating. (**B**) Example of Fourier-space image of excited SPPs. The scale bar is 5 μm. (**C**) Gray image converted from (**B**) and the filed contrast is extracted according to the summation of pixel values inside the left and right regions (red dashed block).

9. Meta-grating Design for Unidirectional Reflection of SPPs

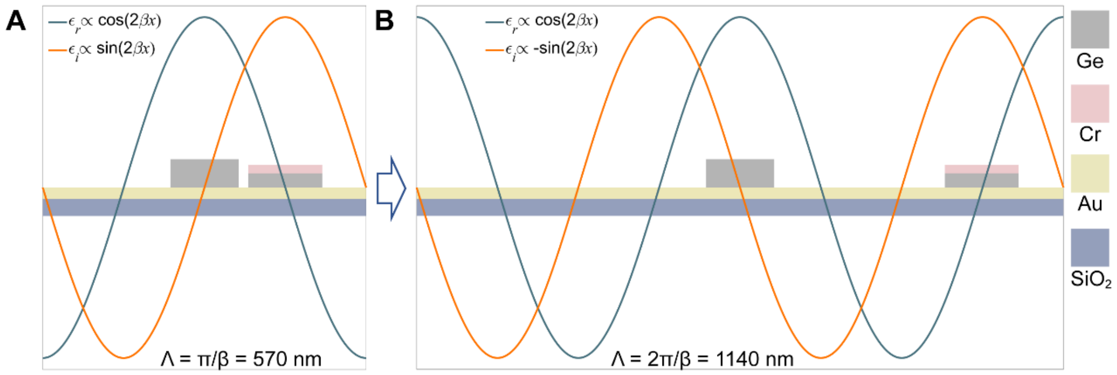

Fig. S10. Optimization of meta-grating design. (**A**) Direct design of the unit cell for directional reflection of SPPs. (**B**) Optimized design of the unit cell for directional reflection of SPPs.

As mentioned in the main text, to achieve asymmetric reflection of SPPs, we need to modify the 2nd order Fourier component of the permittivity profile. We have used a similar combination of nano-strips (with different geometric parameters) in the design. Ideally, we need to shrink the periodicity to half of the wavelength of SPPs, but the separation distance between two nano-

strips is also halved as shown in Fig. S10A. Following a similar approach as the directional excitation case in the previous note, we can optimize the geometric parameters to achieve $A_{2R} \gg A_{2L}$. However, the two nano-strips will be extremely close to each other. It will cause difficulties in fabrications. Therefore, we still use the original periodicity $\Lambda = 1140$ nm, and set the separation distance between nano-strips as $3\Lambda/8$, which inverts the Fourier components (from $\exp(2\beta x)$ to $\exp(-2\beta x)$), as shown in Fig. S10B. As a result, we have a larger separation distance between nano-strips to achieve a reversed Fourier components $A_{2L} \gg A_{2R}$.

10. Characterizing SPPs Reflection

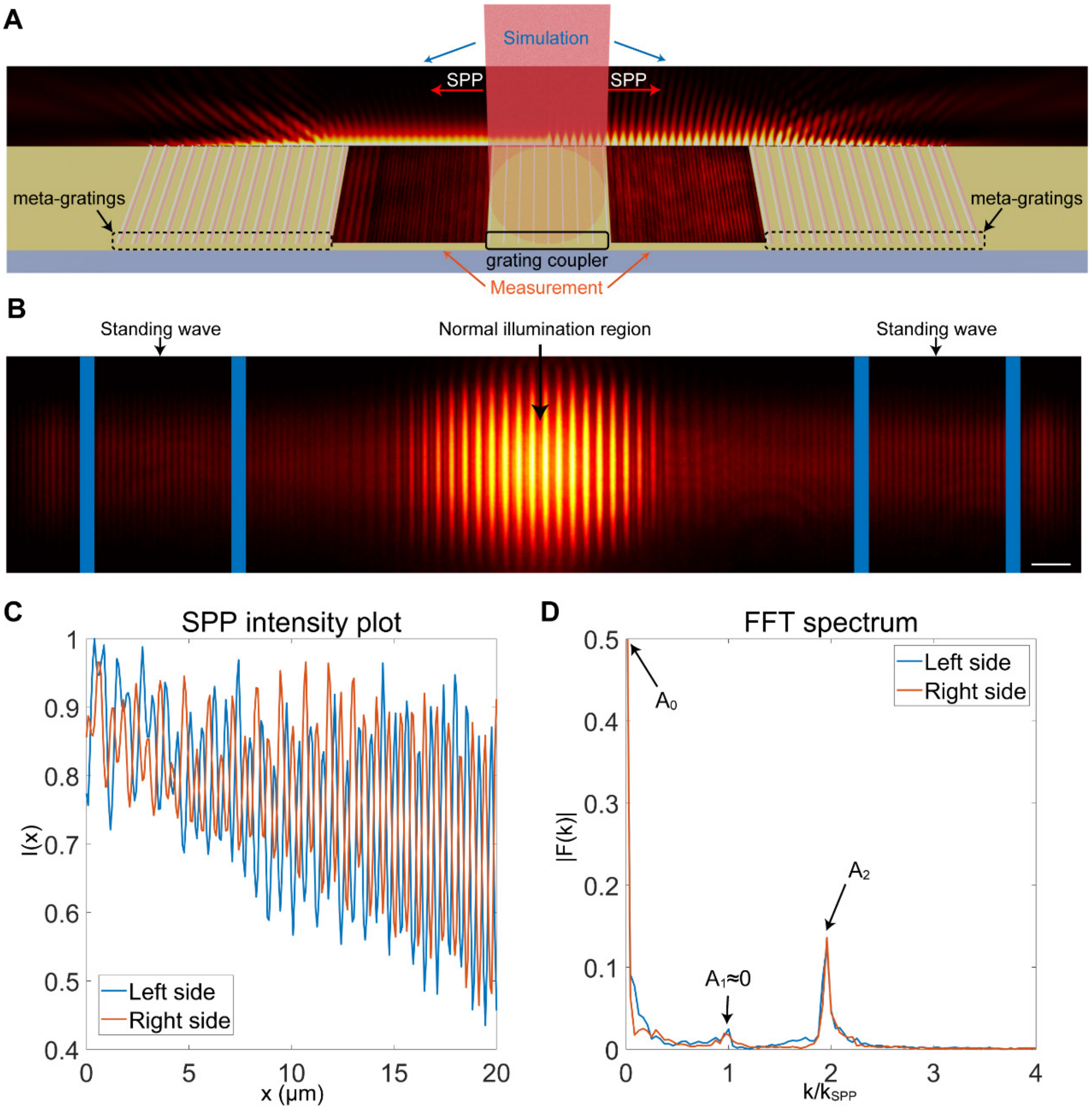

Fig. S11. Method to extract the field contrast of reflected SPPs. (**A**) Schematic of the experimental measurement. (**B**) Example of real-space image of the standing wave of SPPs. The two areas marked by blue lines are the region for image processing. The scale bar is 5 μm. (**C**) The intensity profile along the x-direction is generated from (**B**). (**D**) The Fourier-space components after applying FFT to (**C**).

To measure the reflection of SPPs, we design a grating coupler made of only Ge to evenly excite SPPs to both directions. A schematic of the sample and the experimental setting is presented in Fig. S11A. Two identical meta-gratings are placed 45 μm away from the grating coupler. The SPPs will be reflected by the meta-gratings on both sides, creating a standing wave pattern between the grating coupler and the meta-gratings. We then use the CCD camera to capture the entire 2D plane between the meta-gratings, including the illumination region and the standing wave patterns. One image taken from the measurement is shown in Fig. S11B.

To extract the reflection coefficient of the SPPs from the measured standing wave patterns, we select two symmetric regions in the images with respect to the center of the incident laser spot (also the center of the grating coupler), as indicated by the blue solid lines in Fig. S11B. We convert the image into the gray scale, and then we sum the intensity along the vertical direction of the image. The one-dimensional plot of the interference pattern is shown in Fig. S11C. By applying Fast Fourier Transformation (FFT) to the two intensity plots, we can obtain the Fourier components of the left reflection and right reflection cases, as shown in Fig. S11D.

Since the loss of SPPs at the wavelength of 1150 nm is relatively low (the decay length is about 100 μm according to the measured intensity of SPPs in our experiments), the incident SPPs can be described as $A\exp(-i\beta x)$. If we denote r as the reflection coefficient, the intensity profile of the superimposed SPP waves is

$$\begin{aligned} I(x) &= |A\exp(-i\beta x) + Ar\exp(i\beta x)|^2 \\ &= A^2[(1+r)^2\cos^2\beta x + (1-r)^2\sin^2\beta x] \\ &= A^2[(1-r)^2 + 4r\cos^2\beta x] = A^2[(r^2+1) + 2r\cos(2\beta x)] \\ &\equiv A_0 + A_2\cos(2\beta x) \end{aligned} \tag{S34}$$

where A_0 and A_2 are the 0th and 2nd order Fourier coefficient, respectively. These two coefficients can be obtained from Fig. S10D.

From Equation (S34), it is straightforward to find that

$$\begin{cases} A_0 = A^2(r^2+1) \\ A_2 = A^2(2r) \end{cases} \Rightarrow r = \frac{A_0}{A_2} - \sqrt{\left(\frac{A_0}{A_2}\right)^2 - 1} \tag{S35}$$

Therefore, we can deduce the reflection coefficient r_L and r_R, when the incident SPP is from the left/right side, and the reflectance is calculated as $R_{L/R} = |r_{L/R}|^2$.

REFERENCES AND NOTES

1. C. M. Bender, S. Boettcher, Real spectra in non-Hermitian Hamiltonians having PT symmetry. *Phys. Rev. Lett.* **80**, 5243–5246 (1998).

2. C. M. Bender, D. C. Brody, H. F. Jones, Must a Hamiltonian be Hermitian? *Am. J. Phys.* **71**, 1095–1102 (2003).

3. W. Wang, L. Q. Wang, R. D. Xue, H. L. Chen, R. P. Guo, Y. Liu, J. Chen, Unidirectional excitation of radiative-loss-free surface plasmon polaritons in PT-symmetric systems. *Phys. Rev. Lett.* **119**, 077401 (2017).

4. C. E. Rüter, K. G. Makris, R. el-Ganainy, D. N. Christodoulides, M. Segev, D. Kip, Observation of parity–time symmetry in optics. *Nat. Phys.* **6**, 192–195 (2010).

5. M. Lawrence, N. Xu, X. Zhang, L. Cong, J. Han, W. Zhang, S. Zhang, Manifestation of PT symmetry breaking in polarization space with terahertz metasurfaces. *Phys. Rev. Lett.* **113**, 093901 (2014).

6. M. C. Rechtsman, Optical sensing gets exceptional. *Nature* **548**, 161–162 (2017).

7. W. Chen, S. Kaya Ozdemir, G. Zhao, J. Wiersig, L. Yang, Exceptional points enhance sensing in an optical microcavity. *Nature* **548**, 192–196 (2017).

8. H. Hodaei, A. U. Hassan, S. Wittek, H. Garcia-Gracia, R. el-Ganainy, D. N. Christodoulides, M. Khajavikhan, Enhanced sensitivity at higher-order exceptional points. *Nature* **548**, 187–191 (2017).

9. J. Wiersig, Enhancing the sensitivity of frequency and energy splitting detection by using exceptional points: Application to microcavity sensors for single-particle detection. *Phys. Rev. Lett.* **112**, 203901 (2014).

10. J.-H. Park, A. Ndao, W. Cai, L. Hsu, A. Kodigala, T. Lepetit, Y. H. Lo, B. Kanté, Symmetry-breaking-induced plasmonic exceptional points and nanoscale sensing. *Nat. Phys.* **16**, 462–468 (2020).

11. S. Assawaworrarit, X. Yu, S. Fan, Robust wireless power transfer using a nonlinear parity-time-symmetric circuit. *Nature* **546**, 387–390 (2017).

12. Y. Xu, J.-H. Jiang, H. Chen, Stable lossless polaritons on non-Hermitian optical interfaces. *Phys. Rev. B* **95**, 041409(R) (2017).

13. J. Luo, J. Li, Y. Lai, Electromagnetic impurity-immunity induced by parity-time symmetry. *Phys. Rev. X* **8**, 031035 (2018).

14. H. Zhao, W. S. Fegadolli, J. Yu, Z. Zhang, L. Ge, A. Scherer, L. Feng, Metawaveguide for asymmetric interferometric light-light switching. *Phys. Rev. Lett.* **117**, 193901 (2016).

15. L. Feng, Y. L. Xu, W. S. Fegadolli, M. H. Lu, J. E. B. Oliveira, V. R. Almeida, Y. F. Chen, A. Scherer, Experimental demonstration of a unidirectional reflectionless parity-time metamaterial at optical frequencies. *Nat. Mater.* **12**, 108–113 (2013).

16. H. Alaeian, B. Baum, V. Jankovic, M. Lawrence, J. A. Dionne, Towards nanoscale multiplexing with parity-time-symmetric plasmonic coaxial waveguides. *Phys. Rev. B* **93**, 205439 (2016).

17. H. Alaeian, J. A. Dionne, Non-Hermitian nanophotonic and plasmonic waveguides. *Phys. Rev. B* **89**, 205439 (2014).

18. G. Harari, M. A. Bandres, Y. Lumer, M. C. Rechtsman, Y. D. Chong, M. Khajavikhan, D. N. Christodoulides, M. Segev, Topological insulator laser: Theory. *Science* **359**, eaar4003 (2018).

19. M. A. Bandres, S. Wittek, G. Harari, M. Parto, J. Ren, M. Segev, D. N. Christodoulides, M. Khajavikhan, Topological insulator laser: Experiments. *Science* **359**, eaar4005 (2018).

20. H. Zhao, X. Qiao, T. Wu, B. Midya, S. Longhi, L. Feng, Non-Hermitian topological light steering. *Science* **365**, 1163–1166 (2019).

21. B. Midya, H. Zhao, L. Feng, Non-Hermitian photonics promises exceptional topology of

light. *Nat. Commun.* **9**, 2674 (2018).

22. X. Ni, D. Smirnova, A. Poddubny, D. Leykam, Y. Chong, A. B. Khanikaev, PT phase transitions of edge states at PT symmetric interfaces in non-Hermitian topological insulators. *Phys. Rev. B* **98**, 165129 (2018).

23. B. Zhen, C. W. Hsu, Y. Igarashi, L. Lu, I. Kaminer, A. Pick, S. L. Chua, J. D. Joannopoulos, M. Soljačić, Spawning rings of exceptional points out of Dirac cones. *Nature* **525**, 354–358 (2015).

24. Q. Song, M. Odeh, J. Zúñiga-Pérez, B. Kanté, P. Genevet, Plasmonic topological metasurface by encircling an exceptional point. *Science* **373**, 1133–1137 (2021).

25. A. Regensburger, C. Bersch, M. A. Miri, G. Onishchukov, D. N. Christodoulides, U. Peschel, Parity-time synthetic photonic lattices. *Nature* **488**, 167–171 (2012).

26. K. G. Makris, L. Ge, H. Türeci, Anomalous transient amplification of waves in non-normal photonic media. *Phys. Rev. X* **4**, 041044 (2014).

27. K. Makris, Transient growth and dissipative exceptional points. *Phys. Rev. E* **104**, 054218 (2021).

28. H. Hodaei, M.-A. Miri, M. Heinrich, D. N. Christodoulides, M. Khajavikhan, Parity-time–symmetric microring lasers. *Science* **346**, 975–978 (2014).

29. C. Hahn, S. H. Song, C. H. Oh, P. Berini, Single-mode lasers and parity-time symmetry broken gratings based on active dielectric-loaded long-range surface plasmon polariton waveguides. *Opt. Express* **23**, 19922–19931 (2015).

30. B. Peng, Ş. K. Özdemir, S. Rotter, H. Yilmaz, M. Liertzer, F. Monifi, C. M. Bender, F. Nori, L. Yang, Loss-induced suppression and revival of lasing. *Science* **346**, 328–332 (2014).

31. Y. D. Chong, L. Ge, A. D. Stone, PT-symmetry breaking and laser-absorber modes in optical scattering systems. *Phys. Rev. Lett.* **106**, 093902 (2011).

32. S. Longhi, PT-symmetric laser absorber. *Phys. Rev. A* **82**, 031801 (2010).

33. B. Baum, H. Alaeian, J. Dionne, A parity-time symmetric coherent plasmonic absorber-amplifier. *J. Appl. Phys.* **117**, 063106 (2015).

34. L. Feng, Z. J. Wong, R.-M. Ma, Y. Wang, X. Zhang, Single-mode laser by parity-time symmetry breaking. *Science* **346**, 972–975 (2014).

35. A. Guo, G. J. Salamo, D. Duchesne, R. Morandotti, M. Volatier-Ravat, V. Aimez, G. A. Siviloglou, D. N. Christodoulides, Observation of PT-symmetry breaking in complex optical potentials. *Phys. Rev. Lett.* **103**, 093902 (2009).

36. S. Xiao, J. Gear, S. Rotter, J. Li, Effective PT-symmetric metasurfaces for subwavelength amplified sensing. *New J. Phys.* **18**, 085004 (2016).

37. N. Nye, A. Halawany, C. Markos, M. Khajavikhan, D. Christodoulides, Flexible PT-symmetric optical metasurfaces. *Phys. Rev. Appl.* **13**, 064005 (2020).

38. F. Yang, C. S. Prasad, W. Li, R. Lach, H. O. Everitt, G. V. Naik, Non-Hermitian metasurface with non-trivial topology. *Nanophotonics* **11**, 1159–1165 (2022).

39. D. N. Basov, M. M. Fogler, F. J. Garcia de Abajo, Polaritons in van der Waals materials. *Science* **354**, eaag1992 (2016).

40. T. Low, A. Chaves, J. D. Caldwell, A. Kumar, N. X. Fang, P. Avouris, T. F. Heinz, F. Guinea, L. Martin-Moreno, F. Koppens, Polaritons in layered two-dimensional materials. *Nat. Mater.* **16**, 182–194 (2017).

41. Z. Cai, Y. Xu, C. Wang, Y. Liu, Polariton photonics using structured metals and 2D materials. *Adv. Opt. Mater.* **8**, 1901090 (2019).

42. L. Li, T. Li, S. M. Wang, C. Zhang, S. N. Zhu, Plasmonic Airy beam generated by in-plane diffraction. *Phys. Rev. Lett.* **107**, 126804 (2011).

43. L. Li, T. Li, S. M. Wang, S. N. Zhu, Collimated plasmon beam: Nondiffracting versus

linearly focused. *Phys. Rev. Lett.* **110**, 046807 (2013).

44. P. L. Stiles, J. A. Dieringer, N. C. Shah, R. P. Van Duyne, Surface-enhanced Raman spectroscopy. *Annu. Rev. Anal. Chem.* **1**, 601–626 (2008).

45. J. N. Anker, W. P. Hall, O. Lyandres, N. C. Shah, J. Zhao, R. P. van Duyne, Biosensing with plasmonic nanosensors. *Nat. Mater.* **7**, 442–453 (2008).

46. P. Berini, I. De Leon, Surface plasmon–polariton amplifiers and lasers. *Nat. Photonics.* **6**, 16–24 (2012).

47. W. Srituravanich, L. Pan, Y. Wang, C. Sun, D. B. Bogy, X. Zhang, Flying plasmonic lens in the near field for high-speed nanolithography. *Nat. Nanotechnol.* **3**, 733–737 (2008).

48. M. L. Brongersma, V. M. Shalaev, The case for plasmonics. *Science* **328**, 440–441 (2010).

49. T. W. Ebbesen, C. Genet, S. I. Bozhevolnyi, Surface-plasmon circuitry. *Phys. Today* **61**, 44–50 (2008).

50. J. Chen, Z. Li, S. Yue, Q. Gong, Efficient unidirectional generation of surface plasmon polaritons with asymmetric single-nanoslit. *Appl. Phys. Lett.* **97**, 041113 (2010).

51. B. Eftekharinia, A. Moshaii, A. Dabirian, Design of a slit-groove coupler for unidirectional excitation of the guided surface plasmon polaritons through a plasmonic slot waveguide. *Plasmonics* **12**, 131–138 (2017).

52. K. M. McPeak, S. V. Jayanti, S. J. P. Kress, S. Meyer, S. Iotti, A. Rossinelli, D. J. Norris, Plasmonic films can easily be better: Rules and recipes. *ACS Photonics* **2**, 326–333 (2015).

53. F. J. Rodríguez-Fortuño, G. Marino, P. Ginzburg, D. O'Connor, A. Martínez, G. A. Wurtz, A. V. Zayats, Near-field interference for the unidirectional excitation of electromagnetic guided modes. *Science* **340**, 328–330 (2013).

54. J. Lin, J. P. B. Mueller, Q. Wang, G. Yuan, N. Antoniou, X. C. Yuan, F. Capasso, Polarization-controlled tunable directional coupling of surface plasmon polaritons. *Science*

340, 331–334 (2013).

55. Y. Liu, S. Palomba, Y. Park, T. Zentgraf, X. Yin, X. Zhang, Compact magnetic antennas for directional excitation of surface plasmons. *Nano Lett.* **12**, 4853–4858 (2012).

56. J. Yang, S. Zhou, C. Hu, W. Zhang, X. Xiao, J. Zhang, Broadband spin-controlled surface plasmon polariton launching and radiation via L-shaped optical slot nanoantennas. *Laser Photonics Rev.* **8**, 590–595 (2014).

57. S. Sun, Q. He, S. Xiao, Q. Xu, X. Li, L. Zhou, Gradient-index meta-surfaces as a bridge linking propagating waves and surface waves. *Nat. Mater.* **11**, 426–431 (2012).

58. H. Lingling, C. Xianzhong, B. Benfeng, T. Qiaofeng, J. Guofan, T. Zentgraf, S. Zhang, Helicity dependent directional surface plasmon polariton excitation using a metasurface with interfacial phase discontinuity. *Light Sci. Appl.* **2**, e70 (2013).

59. M. A. Bandres, M. Segev, Non-Hermitian topological systems. *Phys. Ther.* **11**, 96 (2018).

60. A. Hohenau, J. R. Krenn, A. Drezet, O. Mollet, S. Huant, C. Genet, B. Stein, T. W. Ebbesen, Surface plasmon leakage radiation microscopy at the diffraction limit. *Opt. Express* **19**, 25749–25762 (2011).

61. X. Yang, J. Yao, J. Rho, X. Yin, X. Zhang, Experimental realization of three-dimensional indefinite cavities at the nanoscale with anomalous scaling laws. *Nat. Photonics* **6**, 450–454 (2012).

62. D. I. Yakubovsky, A. V. Arsenin, Y. V. Stebunov, D. Y. Fedyanin, V. S. Volkov, Optical constants and structural properties of thin gold films. *Opt. Express* **25**, 25574–25587 (2017).

63. A. Ciesielski, L. Skowronski, W. Pacuski, T. Szoplik, Permittivity of Ge, Te and Se thin films in the 200–1500 nm spectral range. Predicting the segregation effects in silver. *Mater. Sci. Semicond. Process.* **81**, 64–67 (2018).

64. P. Johnson, R. Christy, Optical constants of transition metals: Ti, V, Cr, Mn, Fe, Co, Ni, and

Pd. *Phys. Rev. B* **9**, 5056–5070 (1974).

65. G. Yoon, I. Kim, S. So, J. Mun, M. Kim, J. Rho, Fabrication of three-dimensional suspended, interlayered and hierarchical nanostructures by accuracy-improved electron beam lithography overlay. *Sci. Rep.* **7**, 6668 (2017).
